# In situ characterization of stem cells-like biomarkers in meningiomas

**DOI:** 10.1186/s12935-018-0571-6

**Published:** 2018-05-25

**Authors:** Hanin Alamir, Mona Alomari, Abdulla Ahmed A. Salwati, Mohamad Saka, Mohammed Bangash, Saleh Baeesa, Fahad Alghamdi, Angel Carracedo, Hans-Juergen Schulten, Adeel Chaudhary, Adel Abuzenadah, Deema Hussein

**Affiliations:** 1 Centre of Innovation for Personalized Medicine, King Abdulaziz University, Jeddah, 21589 Saudi Arabia; 2 King Fahd Medical Research Center, King Abdulaziz University, P.O. Box. 80216, Jeddah, 21589 Saudi Arabia; 3 Division of Neurosurgery, King Abdulaziz University, Jeddah, 21589 Saudi Arabia; 4 Pathology Department, King Abdulaziz University, Jeddah, 21589 Saudi Arabia; 5 Galician Foundation of Genomic Medicine-SERGAS, University of Santiago de Compostela, 15706 Santiago de Compostela, Spain; 6 Center of Excellence in Genomic Medicine, King Abdulaziz University, Jeddah, 21589 Saudi Arabia; 7 Faculty of Applied Medical Sciences, King Abdulaziz University, Jeddah, 21589 Saudi Arabia

**Keywords:** Meningioma, Cancer stem cells, Immunofluorescence, CD133, SOX2, Nestin, Frizzled 9, GFAP, SSEA4, Olig2

## Abstract

**Background:**

Meningioma cancer stem cells (MCSCs) contribute to tumor aggressiveness and drug resistance. Successful therapies developed for inoperable, recurrent, or metastatic tumors must target these cells and restrict their contribution to tumor progression. Unfortunately, the identity of MCSCs remains elusive, and MSCSs’ in situ spatial distribution, heterogeneity, and relationship with tumor grade, remain unclear.

**Methods:**

Seven tumors classified as grade II or grade III, including one case of metastatic grade III, and eight grade I meningioma tumors, were analyzed for combinations of ten stem cell (SC)-related markers using immunofluorescence of consecutive sections. The correlation of expression for all markers were investigated. Three dimensional spatial distribution of markers were qualitatively analyzed using a grid, designed as a repository of information for positive staining. All statistical analyses were completed using Statistical Analysis Software Package.

**Results:**

The patterns of expression for SC-related markers were determined in the context of two dimensional distribution and cellular features. All markers could be detected in all tumors, however, Frizzled 9 and GFAP had differential expression in grade II/III compared with grade I meningioma tissues. Correlation analysis showed significant relationships between the expression of GFAP and CD133 as well as SSEA4 and Vimentin. Data from three dimensional analysis showed a complex distribution of SC markers, with increased gene hetero-expression being associated with grade II/III tumors. Sub regions that showed multiple co-staining of markers including CD133, Frizzled 9, GFAP, Vimentin, and SSEA4, but not necessarily the proliferation marker Ki67, were highly associated with grade II/III meningiomas.

**Conclusion:**

The distribution and level of expression of CSCs markers in meningiomas are variable and show hetero-expression patterns that have a complex spatial nature, particularly in grade II/III meningiomas. Thus, results strongly support the notion of heterogeneous populations of CSCs, even in grade I meningiomas, and call for the use of multiple markers for the accurate identification of individual CSC subgroups. Such identification will lead to practical clinical diagnostic protocols that can quantitate CSCs, predict tumor recurrence, assist in guiding treatment selection for inoperable tumors, and improve follow up of therapy.

**Electronic supplementary material:**

The online version of this article (10.1186/s12935-018-0571-6) contains supplementary material, which is available to authorized users.

## Background

Meningiomas occur in multiple extra-axial locations within arachnoid membranes and are highly frequent compared with other types of central nervous system tumors (CNSTs) [1–3]. Genetic analyses of bulk meningioma tissues identified mutations in several pathways including the phosphoinositide 3-kinase (PI3K) and the G protein-coupled receptor smoothened (SMO) signaling pathways [4–6]. Histopathologically, these tumors are classified by the World Health Organization (WHO) into 15 variants within grades I to III. Unfortunately, up to 20% of grade I tumors reoccur, and apart from Mib-1, molecular markers that enable prediction of recurrence have not been established [3, 7, 8].

Meningiomas have been shown to harbor cancer stem cells (CSCs), highly resilient cancer cells that employ deregulated stem cell (SC) expression profiles and are capable of causing reoccurrence [9–14]. Targeting CSCs is predicted to enhance therapy outcomes [3]. A range of genes and their proteins have been associated with the identity of CNST CSCs. CD133/Prominin-1, a five-transmembrane glycoprotein, is normally expressed in embryonic neural SC radial glial/ependymal cells and in ependymal cells in the adult brain [15]. The protein is thought to interact with selected gangliosides to modulate cell-to-cell contact in a cell cycle-related manner [16, 17]. In CNSTs, high CD133 expression has been associated with poor survival [18–21]. In meningioma cell lines, higher CD133 expression correlates positively with cell proliferation and drug resistance [9, 13, 22, 23].

The expression of Nestin, a type VI intermediate filament, has been shown to be important CSC marker for CNST growth, migration, and invasion [24–26], possibly by influencing the cell cycle [27]. Higher expression of Nestin has been detected in grades II and III meningiomas compared to grade I [28]. The deregulated expression of the transcription factor SOX2 has also been observed in several CNST CSCs [29–32]. The knockdown of SOX2 was shown to slow the growth and proliferation of GBM CSCs [33]. In GBM cells positive for CD133, silencing SOX2 impaired tumor initiation and drug resistance [34]. Frizzled 9 (FZD9) belongs to the frizzled protein family, trans-membrane signaling molecules that act as receptors for the WNT protein, and plays a key role in cell development by maintaining planer cell polarity [35]. Mutations in FZD/WNT genes are linked to several malignancies [36]. In astrocytoma and glioblastoma, FZD9 is predominantly expressed by neoplastic cells, and its expression is positively correlated with WHO grading and Ki-67 positivity [37]. Inhibiting the FZD family in glioblastoma cell lines leads to increased differentiation [38].

Stage-specific embryonic antigen-4 (SSEA4), also known as FUT4 and CD15, is a glycosphingolipid (GSL) containing a terminal sialic acid residue (*N*-acetylneuraminic acid) and is involved in the globo-series ganglioside synthesis. SSEA4 is highly expressed during the preimplantation stage in germ cells in the testis and ovaries, and is down-regulated upon differentiation [39–41]. Targeting SSEA4 in vitro suppressed the growth of GBM cell lines [42], and cells positive for SSEA4 have a higher capability for metastasis and invasion [43–47]. Olig2 is a basic helix–loop–helix (bHLH) transcription factor that is expressed in oligodendrocytes and in oligodendritic progenitor cells [48–50]. The protein was shown to mediate the proliferation, migration, and invasion of both normal astrocytes and malignant GBM cells [50–53].

Proteins associated with the differentiation of SCs include Vimentin, glial fibrillary acidic protein (GFAP), and beta III tubulin (βIII-tubulin/βIIIT). Vimentin is a class III intermediate protein that is expressed in mesenchymal cells. The protein’s main function is to support the cytoskeleton [54], and it is highly associated with meningiomas [55]. GFAP is a class III intermediate filament protein, with five different isoforms (GFAPα, GFAPβ, GFAP gamma γ, GFAP δ, and GFAP k), and was shown to be expressed in the astrocyte lineage during the development of the CNS [56, 57]. βIII-Tubulin is a neuron-specific microtubule required for neuronal axon guidance, maintenance, and development [58]. Mutations in the βIII-tubulin gene result in multiple disorders of the CNS [59], and high protein expression is frequently detected in several CNSTs [60]. Although not limited to the identity of CSCs, these markers are frequently associated with it, and their expressions vary according to tumor type and progression [61]. Importantly, recent evidence has indicated that the hetero-identity of CSCs can be detected even within a single tumor developed in a patient [62, 63].

Previously, we published gene expression profiles for most of the meningioma patients’ tissues collected for our cohort [64, 65], as well as for their corresponding cell lines [22]. For this work, we aimed to determine the hetero-dynamic characteristics of MCSCs in situ and identify differential patterns associated with grades II/III tumors.

## Methods

### Sample collection

Meningioma specimens collected between February 2013 and December 2015 were obtained within 30 min of tumor removal and frozen immediately at − 80 °C. Neuropathologists diagnosed surgical specimens according to WHO classification. The clinical profiles for the included patients and their tumors’ histopathological features are shown in Additional file 1: Table S1. Additional file 2: Figure S1 shows H&E representative sections of histological variants of meningiomas included in this work, as well as atypical features. The expression profiles for prevalent cancer driver genes [66], extracted from aforementioned publications, are shown in Additional file 3: Table S2.

### Cyrovial sectioning

Each frozen tissue was cryosectioned to generate 10 consecutive sections at a thickness of 4 µm. Slides of sections were stored at − 20 °C until processed for immunofluorescence.

### Immunofluorescence staining

Sections were left at room temperature for 5 min to defrost, and tissues were enclosed with wax to retain solutions. Then, they were washed five times for 5 min in phosphate buffered saline (PBS). Sections were fixed with 4% formalin for 10 min, then washed three times for 5 in with PBS. Sections were permeabilized, blocked for non-specific antigens with freshly made blocking reagent (5% normal goat serum, 0.25% Triton X-100 in PBS), and incubated for 1 h at room temperature. Single or double primary antibodies solutions (Antibodies, 2% NGS, 0.25% Triton X-100 in PBS) were added to each section, and sections were incubated in a humidity chamber over night at 4 °C. The following day, sections were washed three times for 10 min with 0.25% Triton X-100 in PBS (PBST) before incubating them with a secondary antibodies solution (488 goat anti-mouse (1:300, ab150105, abcam) and 555 goat anti-rabbit (1:700, ab150074, abcam) for 1 h in the dark at room temperature. Sections were then washed five times for 5 min with PBST. PBST was removed, and a drop of Vectashield with DAPI was added to each section to stain nuclei. For each tissue, sections were stained in the following order: secondary only (negative control); mouse anti-Nestin (1:50, ab6142, abcam) with rabbit anti-Ki67 (1:200, ab16667, abcam); mouse anti-CD133 (1:100, 130-092-395, Miltenyi) with rabbit anti-SOX2 (1:200, 09-0024, Stemgent); mouse anti-Vimentin (1:100, ab8978, abcam) with rabbit anti-Frizzled 9 (1:100, ab150515, abcam); rabbit anti-GFAP (1:500, ab7260, abcam); rabbit anti-beta III Tubulin (1:500, ab18207, abcam), mouse anti-SSEA4 (1:100, ab16287, abcam) with rabbit anti-SOX2 (1:200, 130-095-636, Miltenyi); and mouse anti-SSEA4 (1:100, ab16287, abcam) with rabbit anti-Olig2 (1:500, Ab42453, abcam). Processed slides were stored at 4 °C.

### Image acquisition, enhancement, and counting

All images were taken within the first 2 weeks after staining. For each section, five coordinate-fixed dispersed regions were selected to image. Pictures were taken at 20× magnifications using a Leica DMI6000 microscope and Leica DFC425 camera. Photos for individual channels were combined in Photoshop 7.0.1. Enhancements of the images were constrained by signal levels of negative controls of secondary antibodies only. Due to the complexity of staining features, co-positive, mono-positive, and negative cells were manually counted for each region within each section using Photoshop 7.0.1. Manual counting was performed twice by two independent scientists, and indications for positivity for each marker and final counts were confirmed with a neuropathologist. Images for Ki67 stained sections were also counted by an independent third person using automated counting in Image J software for analysis. Images were masked to count nuclei positive for Ki67, and counts were produced using ICTN plugin.

### Statistical analysis of the data

The results were analyzed using SPSS version 21.0 to generate descriptive and inferential statistics. The differences between the manual and automated counts for Ki67 were analyzed using t-tests. The differences for the counts of expressions between grades and the differences in the number of identified unique sub-regions between individual tumors were explored using analysis of variance (ANOVA) robust tests of equality of means, and P-values for Welch and Brown–Forsythe were indicated. Correlations for markers’ expressions across consecutive tumor sections were analyzed using Spearman’s Rho correlation. Chiχ^2^ was used to test for the significance between grades for individual sub-regions.

## Results

### In situ features of SC associated markers in meningiomas

**Fig. 1:**
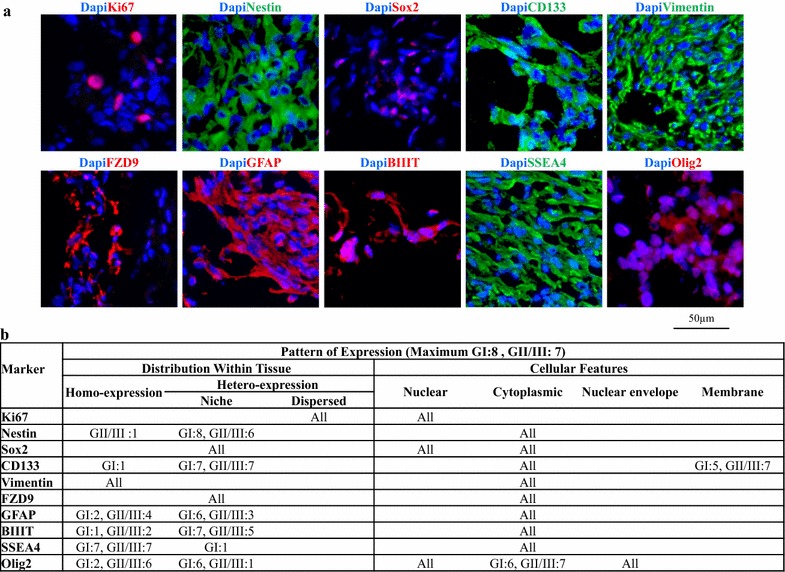
Cellular features and patterns of expression for all the markers used to stain meningioma tissues. **a** Immunofluorescence representative images showing Ki67 (Red), Nestin (green), SOX2 (red), CD133 (green), Vimentin (green), FZD9 (red), GFAP (red), BIIIT (red), SSEA4 (green), and Olig2 (red), each with DAPI (blue). **b** A table summarizing patterns of expression in terms of the distribution within tissue and observed cellular features. *G* grade. All images were taken at ×20

The patterns of expressions for all utilized markers were observed in meningioma tissues (Fig. 1). Positively stained cells for nuclear Ki67 were consistently dispersed as single cells within individual tumor sections. Cells positive for nuclear SOX2 and cytoplasmic FZD9 were consistently seen in niche-stained foci, while cells positive for cytoplasmic Vimentin were detected in large positive regions and had homo-expression patterns. Cells positive for Nestin, CD133, GFAP, BIIIT, SSEA4, and Olig2 had a tumor-dependent pattern of expression, which did not have a dichotomous association with grade. Membranous CD133 was detected in 12 tumors, and Olig2 could be seen at the nuclear envelope, as well as the nucleus, in all tumors.

### Evaluation of the average expressions for single proteins in grade I and grade II/III meningiomas identified GFAP and FZD9 as significant differential markers

**Fig. 2:**
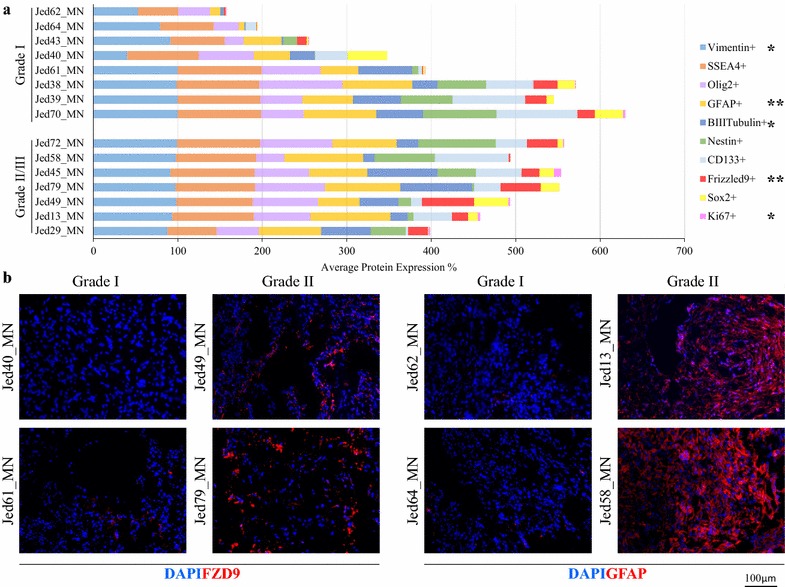
The level of expression for the selected markers in grade I and grade II/III meningioma samples. **a** The average percentages of cells positive for each maker in grade I and grade II/III meningiomas. Significant changes at 0.05 are indicated by * and at 0.01 are indicated by **. **b** Immunofluorescence images for FZD9 and GFAP in a selection of grade I and grade II/III meningiomas. DAPI (blue) FZD9 (red), GFAP (red). Five independent regions were scored for each marker within a stained tumor section. All images were taken at ×20

**Fig. 3:**
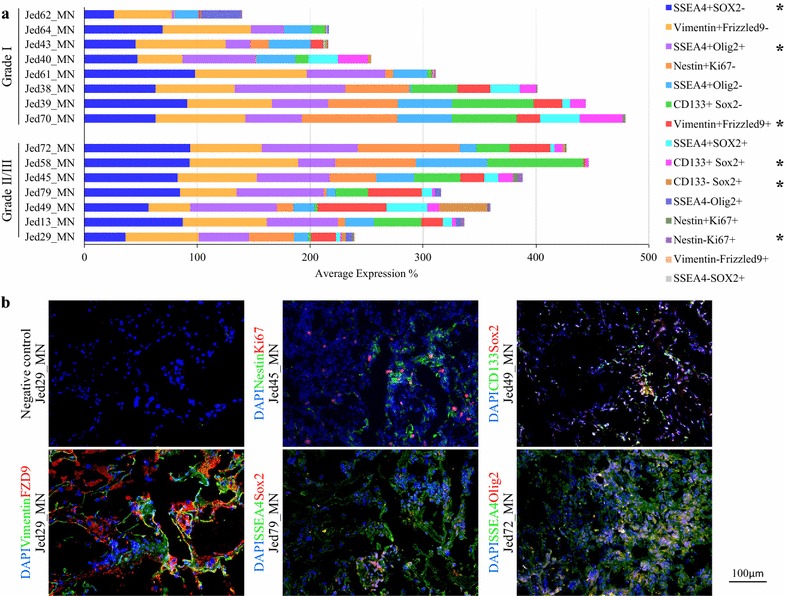
The level of expression for double stained tissues of grade I and grade II/III meningioma samples. **a** The average percentages of cells positive for co-stained markers. Significant changes at 0.05 are indicated by Asterisk. **b** Representative immunofluorescence images for double stained markers for Ki67 (red) with Nestin (green), SOX2 (red) with CD133 (green), Vimentin (green) with FZD9 (red), SSEA4 (green) with SOX2 (red), and SSEA4 (green) with Olig2 (Red), each with DAPI (blue). Five independent regions were scored for each double marker within a stained tumor section. All images were taken at ×20

Data for Ki67 counts showed no significant difference between the manual and automated method (*T* test, P = 0.5), Additional file 4: Figure S2, supporting the use of manual counting for other markers that were complex to assess using automate methods. The analysis of average counts for each single marker’s positive staining for grade I and grade II/III tumors indicated Ki67+, Vimentin+, BIIITubulin+ as differential markers (Brown–Forsythe ANOVA, P < 0.05), respectively, as shown in Table 1 and Fig. 2. For highly significant grade-related differential markers, single positive staining of FZD9+ or GFAP+ was statistically significantly higher in grade II/III meningiomas (Brown–Forsythe ANOVA, P < 0.01). For double-staining analysis (Table 1 and Fig. 3), the most significant average count increase in grade II/III meningiomas was seen for Vimentin+FZD9+ (Brown–Forsythe ANOVA, P < 0.01). The averages for cell count staining SSEA4+Olig2+, Nestin−Ki67+, or CD133−Sox+ were also higher in grade II/III meningiomas (Brown–Forsythe ANOVA, P < 0.05), while the average for the number of CD133+Sox+ cells decreased in grade II/III compared to grade I meningiomas (Brown–Forsythe ANOVA, P < 0.05).

**Table 1:** The means of expressions, standard errors, and ANOVA P values for grade I versus grade II/III tumors for single and double-stained markers

Marker(s)	Grade	Mean	STD error	P value
Nestin+	GI	29.45	5.70	0.231
	GII/III	39.22	5.73	
Ki67+	GI	0.77	0.17	0.019*
	GII/III	2.72	0.78	
CD133+	GI	36.60	6.22	0.770
	GII/III	39.11	5.86	
Sox2+	GI	12.45	2.76	0.929
	GII/III	12.82	3.22	
Vimentin+	GI	83.13	4.31	0.016*
	GII/III	94.56	1.57	
Frizzled9+	GI	11.82	2.35	0.000**
	GII/III	31.23	4.10	
GFAP+	GI	49.11	5.80	0.000**
	GII/III	78.03	3.28	
BIIITubulin+	GI	30.65	5.16	0.033*
	GII/III	47.01	5.49	
SSEA4+	GI	75.70	4.57	0.053
	GII/III	87.11	3.57	
Olig2+	GI	55.33	5.02	0.072
	GII/III	67.55	4.43	
Nestin+Ki67+	GI	0.51	0.16	0.080
	GII/III	1.23	0.37	
Nestin+Ki67−	GI	28.94	5.59	0.258
	GII/III	37.98	5.63	
Nestin−Ki67+	GI	0.26	0.08	0.037*
	GII/III	1.49	0.56	
CD133+Sox2+	GI	11.73	2.71	0.039*
	GII/III	5.48	1.20	
CD133+Sox2−	GI	24.87	4.41	0.224
	GII/III	33.63	5.61	
CD133−Sox2+	GI	0.72	0.31	0.023*
	GII/III	7.35	2.77	
Vimentin+FZD9+	GI	11.80	2.36	0.000**
	GII/III	31.08	4.12	
Vimentin+FZD9−	GI	71.34	3.87	0.159
	GII/III	63.48	3.95	
Vimentin−FZD9+	GI	0.03	0.02	0.125
	GII/III	0.15	0.08	
SSEA4+SOX2+	GI	12.57	2.50	0.565
	GII/III	10.59	2.36	
SSEA4+SOX2−	GI	63.13	4.12	0.021*
	GII/III	76.52	3.91	
SSEA4−SOX2+	GI	0.07	0.05	0.539
	GII/III	0.03	0.03	
SSEA4+Olig2+	GI	49.81	5.42	0.035*
	GII/III	64.64	4.29	
SSEA4+Olig2-	GI	29.15	3.51	0.324
	GII/III	23.97	3.85	
SSEA4−Olig2+	GI	5.52	2.95	0.405
	GII/III	2.91	0.95	

P values for ANOVA Welch and Brown–Forsythe are indicated

* P significant at the 0.05 level (2-tailed)

** P is significant at the 0.01 level (2-tailed)

### Consecutive sections have similar expressions for a single marker

**Fig. 4:**
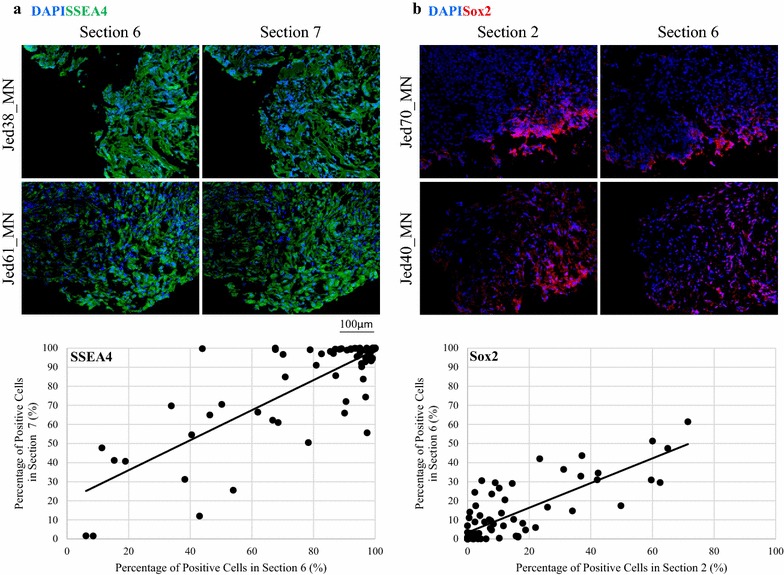
The correlation of the expression of SSEA4 and SOX2 in adjacent and distal consecutively sectioned immunofluorescence-processed tissues. **a** Representative immunofluorescence images for adjacent sections 6 and 7 stained for SSEA4 (green), and for distal sections 2 and 6 stained for SOX2 (red). All images were taken at ×20. **b** Graphs showing Spearman’s Rho correlations between positive expression of SSEA4 in sections 6 and 7 or SOX2 in sections 2 and 7, for all samples

To determine the nature of the positive spatial distribution of a single marker throughout the depth of a tumor, the expression profile for both SSEA4 and SOX2 was determined in adjacent and distal consecutively sectioned immunofluorescence-processed tissues. Adjacent sections six and seven were stained to detect SSEA4, while distal sections two and six were stained to detect SOX2 (Fig. 4). The percentages of cells positive for SSEA4 in section six correlated with positive cells for SSEA4 in the adjacent section seven (Spearman’s Rho correlation coefficient = 0.687, P < 0.001). Similarly, the percentages of cells positive for SOX2 in section two correlated with positive cells for SOX2 in the distal section six (Spearman’s Rho correlation coefficient = 0.749, P < 0.001).

### There are significant correlations between the expressions of different SC associated markers across consecutive tissues

**Fig. 5:**
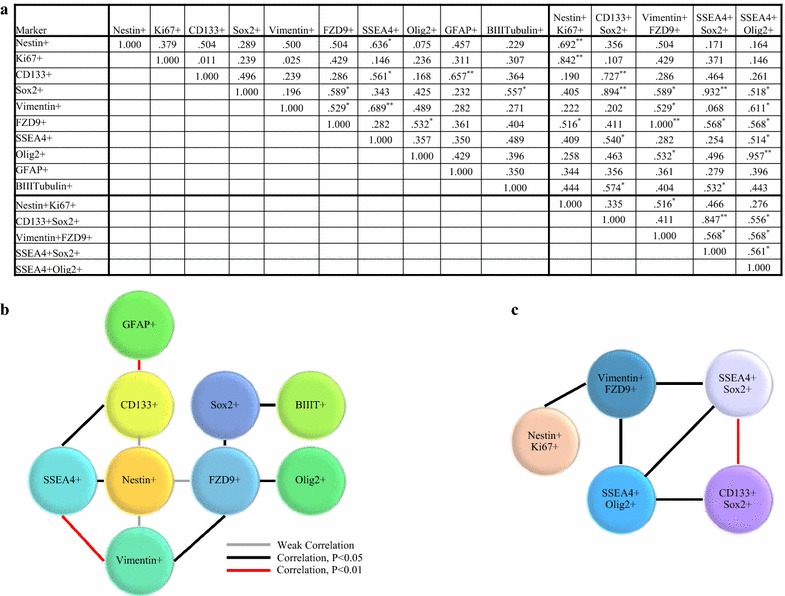
Correlation trends between the expressions of different markers across consecutive tissues. **a** A list showing Spearman’s Rho correlation coefficients. *Correlation is significant at the 0.05 level (2-tailed). **Correlation is significant at the 0.01 level (2-tailed). **b** Illustrations of the strength of correlations between different single markers, and **c** co-stained markers

Since the expression profiles of each of SOX2 and SSEA4 were equivalently spatially distributed throughout consecutive sections of a tumor mass, correlations between the expressions of different single markers across all consecutive sections were investigated (Fig. 5). Expression data indicated a highly significant correlation between the expressions of Vimentin and SSEA4 and the expressions of CD133 and GFAP. Significant correlations were observed for the expressions of SSEA4 with CD133 or Nestin, and SOX2 with BIIIT. FZD9 also had significant correlations with Vimentin, SOX2 or with Olig2. The presence of Nestin-positive proliferating cells correlated with the presence of Vimentin+FZD9+ cells.

### Qualitative analysis of sub-areas across consecutive sections show increased hetero-regional expression in grades II/III meningiomas

**Fig. 6:**
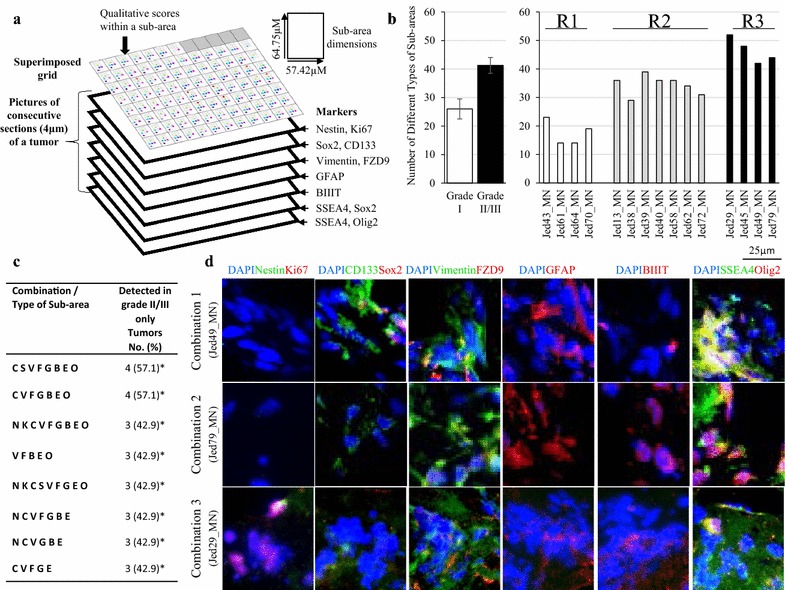
Hetero-regional expression analysis of sub-areas across consecutive sections for all meningioma tissues. **a** A diagram depicting consecutive sectioning and immunofluorescence staining for each section. **b** Bar graphs showing the number of different types of sub-areas for all tumors separated into significantly differential groups (R1, R2, R3) or grades (grade I, grade II/III). **c** Regions that significantly frequently occurred in grade II/III but never in grade I meningiomas. **d** Representative immunofluorescence images for consecutive sections for significantly frequently occurring combinations in grade II/III meningiomas. Sections were double stained for Ki67 (red) with Nestin (green), SOX2 (red) with CD133 (green), Vimentin (green) with FZD9 (red), SSEA4 (green) with SOX2 (red), and SSEA4 (green) with Olig2 (red), each with DAPI (blue). Single staining of GFAP (red) and BIIITubulin (red) is also shown. All images were taken at ×20

To investigate the relationship between multiple markers across consecutive sections, images for a coordinate-fixed region within stained sections were scored using a grid with 96 sub-regions, each covering an area of 0.0037 mm^2^. The grid was used as a repository sheet of qualitative information for positive staining in each sub-area for all consecutive sections of each tumor, as exemplified in Fig. 6a, Additional file 5: Figure S3, and Additional file 6: Figure S4. Collectively, the data showed a complex distribution of the scoring of the combined SC associated markers, across individual tissues (208 unique combinations, Additional file 7: Table S3), with increased hetero-regional expression being associated with grade II/III meningiomas (ANOVA, P < 0.01, Fig. 6b). Interestingly, the level of hetero-regional expression separated tumors into three significantly different groups (ANOVA, P < 0.01), with all tumors in group 1 (R1) being grade I and all meningiomas in group 3 (R3) being grade II/III, while tumors in group 2 (R2) had mixed grades for I and II. Regions that were significantly frequently occurring in grade II/III but never in grade I meningiomas included those that were positive for CD133+SOX2±Vimentin+FZD9+GFAP+BTIII+SSEA4+Olig2+, and Nestin+Ki67+CD133+Vimentin+FZD9+GFAP+BTIII+SSEA4+Olig2+ (Fig. 6c, d).

## Discussion

Collectively, meningiomas present a unique model for exploring tumor progression in CNSTs, as they encompass tumors with a variety of aggressiveness and grades. Our study sheds a light into the protein expression and co-localization of critical SC and developmental markers that are implicated in modulating malignancy. In particular, we present a comprehensive differential analysis of the three dimensional spatial distribution of SC markers in situ, their co-expression, and their correlation in relation to grade.

The features observed for individual proteins in the meningioma samples were consistent with their manufacturing data and previous publications in other tissue types [42, 57, 67–73]. Ki67-positive cells were clearly dispersed, indicating that dividing cells were not particularly grouped together. Both SOX2 and FZD9 were less frequent and occurred in niches, which is in concordant with niche-organized CSCs. All other studied markers had variable characteristics that had either niche, hetero-, or homo-expression, in a tumor-dependent manner. Of particular interest is the localization of Olig2. The exclusion of this protein from the nucleus has been reported to be associated with astrocyte differentiation, while nuclear Olig2 was shown to target chromatin remodelers, prior differentiation in oligodendrocyte progenitors [49, 53, 74]. In this cohort, Olig2 was predominantly observed in the nucleus, at the nuclear envelope, and only occasionally in the cytoplasm, thus implying that meningioma cells may behave like oligodendrocyte progenitors. However, further detailed work is required to clarify this observation and future studies will need to be completed on a larger scale.

Notably, the expression of all individual proteins was not dichotomous for grade. Cells positive for all SC markers were detected in grade I meningiomas, suggesting that either the establishment of CSC clones occurs early in tumor development, or that by the time tumors become clinically evident, CSCs are already established. However, consistent with published data, a higher number of positive cells stained for Ki67 and Vimentin were detected in grade II/III compared with grade I meningiomas [13, 69]. To the best of our knowledge, this study is the first to present in situ analysis of the expression of SSEA4, OLIG2 and FZD9 in meningiomas. Cells positive for SSEA4 and OLIG2 were more frequent in grade II/III meningiomas and the number of FZD9-positive cells was significantly higher in grade II/III meningiomas, although the overall levels remained relatively low, implying that growth of FZD9-positive cells in meningiomas is restricted.

Surprisingly, and in contrast to other studies, more cells positive for GFAP or BIIIT were detected in grade II/III meningiomas [75]. A form of GFAP that differs in the C-terminal domain was detected in the subventricular zone (SVZ) of the brain, suggesting that GFAP may not be an exclusive astrocytic differentiation marker [56, 57]. Indeed, it is important to consider that for proteins with multiple forms, the detection of a protein’s expression using immunostaining will depend on the utilized antibody [76]. According to the manufacturing information sheet, the GFAP antibody used in this work was raised against the full length of a purified native protein corresponding to human GFAP.

Compared to previous studies [10, 13, 28, 67, 68, 77, 78], co-staining for SOX2, CD133 and Nestin across a single section also provided a few unexpected observations. In particular, the average number of cells positive for both SOX2 and CD133 was lower in grade II/III meningiomas, while cells positive for SOX2 and negative CD133 increased in frequency. The increase in the later was particularly noted in the recurrent tumor Jed49_MN. The fraction of Ki67+ cells that were Nestin negative were more frequent in grade II/III meningiomas, even though Nestin expression tended to slightly increase with grade [28]. Together, these observations may be explained by the CSC clonal evolution theory, where for example, cells positive for SOX2 and CD133 could occur at early development and diverge later to partner with other SC-related genes [79]. In addition, they highlight in vitro and in situ differences in the expression of CSCs markers that may reflect epigenetic changes, influenced by the microenvironment.

The analysis of a single marker throughout the consecutive sections along a depth of 32 μm indicated a strong correlation of expression for both adjacent and distal sections of meningioma tissues. Basic analysis locating CSC niches across consecutive sections has been attempted previously in breast cancer tissues [80, 81]; however, no correlation of expression was studied. Spearman’s Rho factor indicated a highly significant correlation between the expressions of Vimentin and SSEA4, and the expressions of CD133 and GFAP. The co-expression of SSEA4 and Vimentin has been observed in multipotent mesenchymal SCs and in postnatal periodontal ligament (PDL)-derived SCs (PDLSC) [11, 82]. CD133 and GFAP co-expression has been detected in glioneuronal tumors [83], glioblastoma cells [84], and activated B1 astrocytes [85, 86]. Such correlation implicates activated B1 astrocytes’ expression-like program in at least a fraction of meningioma cells. Significant correlations were also observed for the expressions of SSEA4 with CD133 or Nestin, FZD9 with Vimentin or SOX2 or Olig2, and SOX2 with BIIIT. Enrichment for SSEA4 and CD133-positive cells from cord blood marked very small embryonic-like stem cells (VSELs) that have high telomerase activity and express pluripotent SC markers OCT4, SSEA4, NANOG, and SOX2 [87]. Similarly, the co-expression of SSEA4 and Nestin has been observed in human umbilical cord matrix-derived mesenchymal SCs [88]. The presence of Nestin-positive proliferating cells also correlates with the presence of Vimentin+FZD9+ cells. Co-expression of FZD9 and Nestin has been observed in neural stem progenitor, derived from patients with Williams syndrome, a developmental disorder caused by mutations in chromosome 7 [89]. The correlation of FZD9 with SOX2 is perhaps not surprising, giving that they are both part of the WNT signaling pathway, a pathway that is activated in some meningiomas [37]. Perhaps more surprising is the correlation between SOX2 and BIIIT. This combination has been implicated in taxane resistance for patients with stage III ovarian epithelial cancer [90] and observed in GBM cell lines [91]. Interestingly, the expression of Ki67 alone does not correlate with any particular marker, suggesting that proliferating cells belong to a heterogeneous population of clones. Alternatively, cells may be exiting SC-like status to divide.

An increase in the tumor heterogeneity of CNSTs has long been associated with aggressiveness, resistance, and reoccurrence [79, 92–96]. Recent studies have addressed heterogeneity using novel and challenging approaches [62, 97]; however, very few are documented for meningiomas. In situ analysis can harness the spatial information of tumor heterogeneity [98, 99], in particular, the analysis of consecutive sections that provide three dimension spatial information. While the association of CSCs heterogeneity in CNSTs with high tumor aggressiveness is currently being debated [100, 101], the data presented here show a clear difference in the hetero-regional expression of the investigated markers for grade I and grade II/III meningiomas. Interestingly, however, hetero-regional expression could be detected even in grade I tumors. In addition, particular combinations occur frequently in grade II/III and never in grade I meningiomas. Both observations could be explained by the CSC evolution hypothesis, where CSCs acquire new changes in the early development of disease and continue to acquire new changes with progressive disease [23, 95]. Whether the identified combinations detected only in grade II/III meningiomas can be used for predictive diagnostic purpose remains to be seen, as a larger cohort of high grade meningiomas is needed. Nevertheless, these results highlight that similarly to neural SC markers [85], and due to CSC heterogeneity, markers must be used in combinations to ensure proper CSC identification. Any aspirations to develop targeted therapies for CSCs are dependent on accurate identifications of all heterogeneous populations.

## Conclusion

Meningiomas present a unique human model for exploring CSCs progression in CNSTs, as they encompass a variety of tumors that differ in growth rates and the capacity to reoccur or metastasize. Using a potentially widely applicable method for analyzing consecutive sections, our study presents a comprehensive differential analysis of the three dimensional spatial distribution of CSC markers, their co-expression, and their correlation in relation to grade. The distribution and the level of expression for individual CSCs markers in meningiomas are variable between patients, however, collective analysis of markers indicates a complex spatial nature that is particularly associated with higher grades. Thus, results strongly support the notion of heterogeneous populations of CSCs, even in grade I meningiomas, and call for the use of multiple markers for the accurate identification of individual CSC subgroups. Such identification will lead to practical clinical diagnostic protocols that can quantitate CSCs, predict tumor recurrence, assist in guiding treatment selection for inoperable tumors, and improve follow up of therapy.

## Additional files

**Additional file 1: Table S1.** The clinical profiles for the included patients and their tumors’ histopathological features.

**Additional file 2: Figure S1.** H&E images for different morphological variants and atypical features for meningiomas used in this study. A. Images showing WHO classified morphological features for different meningioma variants. Meningothelial (Jed39_MN) with neoplastic growth of syncytial epithelial cells with indistinct cell borders arranged in whorls; fibroblastic (Jed40_MN) showing spindle cells with indistinct cell boundaries running in fascicle; transitional (Jed38_MN) with ratios of meningothelial to fibroblastic patterns 40:60; psammomatous (Jed43_MN) composed of whorled clusters of spindle cells with numerous psammoma bodies; chordoid (Jed79_MN), Cords of epithelioid cells with focal clear to foamy cytoplasm on myxoid stroma.; rhabdoid (Jed29_MN) showing hypercellular sheets with rhabdoid morphology. B. Tumors with atypical features. Images show patternless growth (sheeting) in Jed72_MN, necrosis and small cells with high nuclear to cytoplasm ratio in Jed58_MN, and brain invasion in Jed13_MN. Magnifications are indicated above images.

**Additional file 3: Table S2.** Differentially expressed cancer driver genes [66] in individual tumors compared with three normal brain sample data sets, referenced in Gene Expression Omnibus (GEO) submission GSE77259. Values were generated from previously published data sets [64, 65] using Transcriptome Analysis Console v. 4.0.

**Additional file 4: Figure S2.** A bar graph showing averages of counts for Ki67 stained sections collected using manual counting or automated counting in Image J software.

**Additional file 5: Figure S3.** A. Representative immunofluorescence images for consecutive sections for the grade I Jed64_MN meningioma. Sections were double stained for Ki67 (red) with Nestin (green), SOX2 (red) with CD133 (green), Vimentin (green) with FZD9 (red), SSEA4 (green) with SOX2 (red), and SSEA4 (green) with Olig2 (red), and each section was stained with DAPI (blue). Single staining of GFAP (red) or BIIITubulin (red) is also shown. All images were taken at 20x. B. A grid used as a repository of information for categorical staining is shown with a color-coded legend and size dimensions for sub-areas.

**Additional file 6: Figure S4.** A. Representative immunofluorescence images for consecutive sections for the grade III Jed29_MN meningioma. Sections were double stained for Ki67 (red) with Nestin (green), SOX2 (red) with CD133 (green), Vimentin (green) with FZD9 (red), SSEA4 (green) with SOX2 (red), and SSEA4 (green) with Olig2 (red), and each section was stained with DAPI (blue). Single staining of GFAP (red) or BIIITubulin (red) is also shown. All images were taken at 20x. B. A grid used as a repository of information for categorical staining is shown with a color-coded legend and size dimensions for sub-areas.

**Additional file 7: Table S3.** All combinations of markers observed in consecutive sections and their frequencies in all 15 meningioma samples.

## Abbreviations

CNSTs: central nervous system tumors
WHO: World Health Organization
CSCs: cancer stem cells
KAUH: King Abdulaziz University Hospital
FZD9: Frizzled9
GFAP: glial fibrillary acidic protein
SSEA4: specific embryonic antigen-4
βIII-tubulin/βIIIT: beta III tubulin
PIK3: phosphoinositide 3-kinase
SMO: G protein-coupled receptor smoothened
GSL: glycosphingolipid
PDMP: phenyl-2-decanoylamino-3-morphilino-1-propranol
bHLH: basic helix–loop–helix
PBS: phosphate buffered saline
PBST: Triton X-100 in PBS
NGS: normal goat serum
SPSS: Statistical Analysis Software Package
ANOVA: analysis of variance
SVZ: subventricular zone
PDLSC: postnatal periodontal ligament (PDL)-derived SC
VSELs: very small embryonic-like stem cells
