# Supplementary material for: In situ characterization of stem cells-like biomarkers in meningiomas
Source: Cancer Cell Int. 2018 May 25;18:77. doi: 10.1186/s12935-018-0571-6 (PMC5970464; doi:10.1186/s12935-018-0571-6)
Supplement: Supplementary file 1 — Additional file 1: Table S1. The clinical profiles for the included patients and their tumors’ histopathological features. [file 12935_2018_571_MOESM1_ESM.docx]

**Additional Table 1.** **The clinical profiles for the included patients and their tumors’ histopathological features.**

| **Tumor** | **Age** | **Gender** | **Sub-classification** | **Grade** | **P/R** | **Tumor site** | **Summary of Histological Features** | **Treatment** |
| --- | --- | --- | --- | --- | --- | --- | --- | --- |
| Jed13_MN | 50 | F | Fibroblastic  (Atypical meningioma) | II | P | Supratentorial | Neoplastic proliferation growth of spindle cells arranged in interlacing fascicles with bland- looking cells. Some atypical features present (grade II): Areas of zonal necrosis, prominent nucleoli, increased cellularity and brain invasion. Other feature: 3 mitotic figures/ 10 high power field (HPF). | Surgery 39706-1 |
| Jed29_MN | 32 | M | Rhabdoid | III | ^2nd^ R | Right middle fossa | Hypercellular neoplastic growth arranged into solid sheets of cells with rhabdoid morphology: eccentric pleomorphic nuclei and abundant eosinophilic cytoplasm. Mitotic activity: 16 mitosis/ 10 HPF. Several areas of necrosis. WHO grade III by type (Rhabdoid meningioma). | Surgery 9007-02 |
| Jed38_MN | 46 | F | Transitional | I | P | Left clinoidal | Biphasic neoplastic growth: spindle fibroblast-like cells and epithelial cells with indistinct cell borders arranged in whorls. Few Psammoma bodies. Mitotic activity: 0 mitosis/ 10 HPF. No atypical features*. No brain tissue present. | Surgery 39706-1 |
| Jed39_MN | 33 | F | Meningothelial | I | P | Left subfrontal | Neoplastic growth of syncytial epithelial cells with indistinct cell borders arranged in whorls. Many psammoma bodies. Mitotic activity: 0 mitosis/ 10 HPF. No atypical features. No brain tissue present. | Surgery 9007-02 |
| Jed40_MN | 64 | F | Fibroblastic | I | P | Cerebellopontine angle | Neoplastic growth composed of spindle shaped cells arranged in fascicles and storiform. Psammomatous bodies are seen. Mitotic activity: 0 mitosis/ 10 HPF. No atypical features. No brain tissue present. Ki67 index is less than 5%. Immunohistochemistry shows EMA focally positive, Vimentin +, S100 +, GFAP -. | Surgery 9007-02 |
| Jed43_MN | 55 | F | Psammomatous | I | P | Extra axial spinal | Neoplastic growth composed mainly of numerous psammoma bodies. Few clusters of spindle cells and syncytial epithelial cells arranged in whorled. Mitotic activity: 0 mitosis/ 10 HPF. No atypical features. No brain tissue present. | Surgery 40312-00 |
| Jed45_MN | 33 | M | Metastatic Rhabdoid | III | 3^rd^ R | Vertebral body | Fibrocollagenous tissue mixed with bone trabeculae infiltrated by malignant cells arranged in sheets and exhibiting high nuclear to cytoplasm ratio, pleomorphism, hyperchromatic eccentric nuclei with prominent nucleoli. WHO grade III by type (Rhabdoid meningioma). Immunohistochemistry shows diffuse PR weakly positive, Cytokeratin 7 -, P63 - . | Prior excision treatment of Carboplatin (3x 800 mg) & Radiotherapy. 48639-00 Excision of vertebra. |
| Jed49_MN | 51 | F | Fibroblastic  (Atypical meningioma) | II | R | Superior sagittal sinuses | Neoplastic growth composed of spindle shaped cells arranged in interlacing fascicles. Foci of Psammoma bodies are seen. Necrosis was noted, no other atypical features. Mitotic activity: 4 mitosis/ 10 HPF. | Surgery 39703-02 |
| Jed58_MN | 47 | M | Atypical meningioma | II | P | Right frontal extra axial | Neoplastic growth of meningiothelial cells with many atypical features: increased cellularity, small cells with high N/C ratio, large and prominent nucleoli, patternless growth and foci of spontaneous necrosis. Mitotic activity: 5 mitosis/ 10 HPF | Surgery 39706-1 |
| Jed61_MN | 48 | F | Fibroblastic | I | P | Intra axial right frontal | Neoplastic meningiothelial proliferation of spindle cells arranged in interesting fascicles with bland- looking cells. Mitotic activity: 0 mitosis/ 10 HPF. No atypical features. No brain tissue present. | Surgery 39706-1 |
| Jed62_MN | 44 | F | Transitional | I | P | Right convexity | Biphasic neoplastic growth: spindle fibroblast-like cells arranged in long fascicules and epithelial cells with indistinct cell borders arranged in whorls. Foci of ischemic necrosis and iatrogenic embolic blood vessels with embolic agents. Atypical features not seen. No brain present. | Excision via burr holes |
| Jed64_MN | 49 | F | Fibroblastic | I | P | Supresellar | Spindle cells arranged mainly in fascicles. Mitotic activity: 0 mitosis/ 10 HPF. No atypical features. No brain tissue present. S100 +, PR +, EMA +. | Surgery 9007-02 |
| Jed70_MN | 73 | F | Fibroblastic | I | P | Right sphenoid wing | Spindle cells arranged mainly in fascicles. Mitotic activity: 0 mitosis/ 10 HPF. No atypical features. No brain tissue present. EMA +, Vimentin + | Surgery 9007-02 |
| Jed72_MN | 31 | F | Atypical meningioma | II | P | Right frontal region | Neoplastic growth arranged mainly in sheets. Infrequent mitotic activity up to 10 mitosis/10 HPF is identified. Multiple nodules of meningioma invading adjacent normal brain tissue is identified. Atypical features namely: increased cellularity, small cells with high N/C ratio, prominent nucleoli, patternless growth and foci of spontaneous necrosis are present. | Surgery 9007-02 |
| Jed79_MN | 19 | M | Chordoid | II | P | Right convexity | Cords of epithelioid cells with focal clear to foamy cytoplasm on myxoid stroma. WHO grade II by type (chordoid type). Vimentin +, S100 -, EMA +, | Surgery 39706-1 |

Surgical treatments codes indicate: 39706-1 Decompression and osteoplastic craniotomy and excision, 9007-02 Craniotomy, other procedure and excision, 397-09-2 Removal of lesion of cerebellum, 39706-1 Decompression and osteoplastic craniotomy and excision, 40312-00 Removal of spinal intradural lesion, 48639-00 Excision of vertebra (prior treatment of carboplatin), 39703-02 Biopsy of cerebral meninges. P/R: Primary/ Recurrent. Atypical features include increased cellularity, small cells with high nuclear to cytoplasmic ratio, prominent nucleoli, pattern-less growth and foci of spontaneous or geographic necrosis.
