# Supplementary material for: In situ characterization of stem cells-like biomarkers in meningiomas
Source: Cancer Cell Int. 2018 May 25;18:77. doi: 10.1186/s12935-018-0571-6 (PMC5970464; doi:10.1186/s12935-018-0571-6)
Supplement: Supplementary file 2 — Additional file 2: Figure S1. H&E images for different morphological variants and atypical features for meningiomas used in this study. A. Images showing WHO classified morphological features for different meningioma variants. Meningothelial (Jed39_MN) with neoplastic growth of syncytial epithelial cells with indistinct cell borders arranged in whorls; fibroblastic (Jed40_MN) showing spindle cells with indistinct cell boundaries running in fascicle; transitional (Jed38_MN) with ratios of meningothelial to fibroblastic patterns 40:60; psammomatous (Jed43_MN) composed of whorled clusters of spindle cells with numerous psammoma bodies; chordoid (Jed79_MN), Cords of epithelioid cells with focal clear to foamy cytoplasm on myxoid stroma.; rhabdoid (Jed29_MN) showing hypercellular sheets with rhabdoid morphology. B. Tumors with atypical features. Images show patternless growth (sheeting) in Jed72_MN, necrosis and small cells with high nuclear to cytoplasm ratio in Jed58_MN, and brain invasion in Jed13_MN. Magnifications are indicated above images. [file 12935_2018_571_MOESM2_ESM.pptx]

## Slide 1
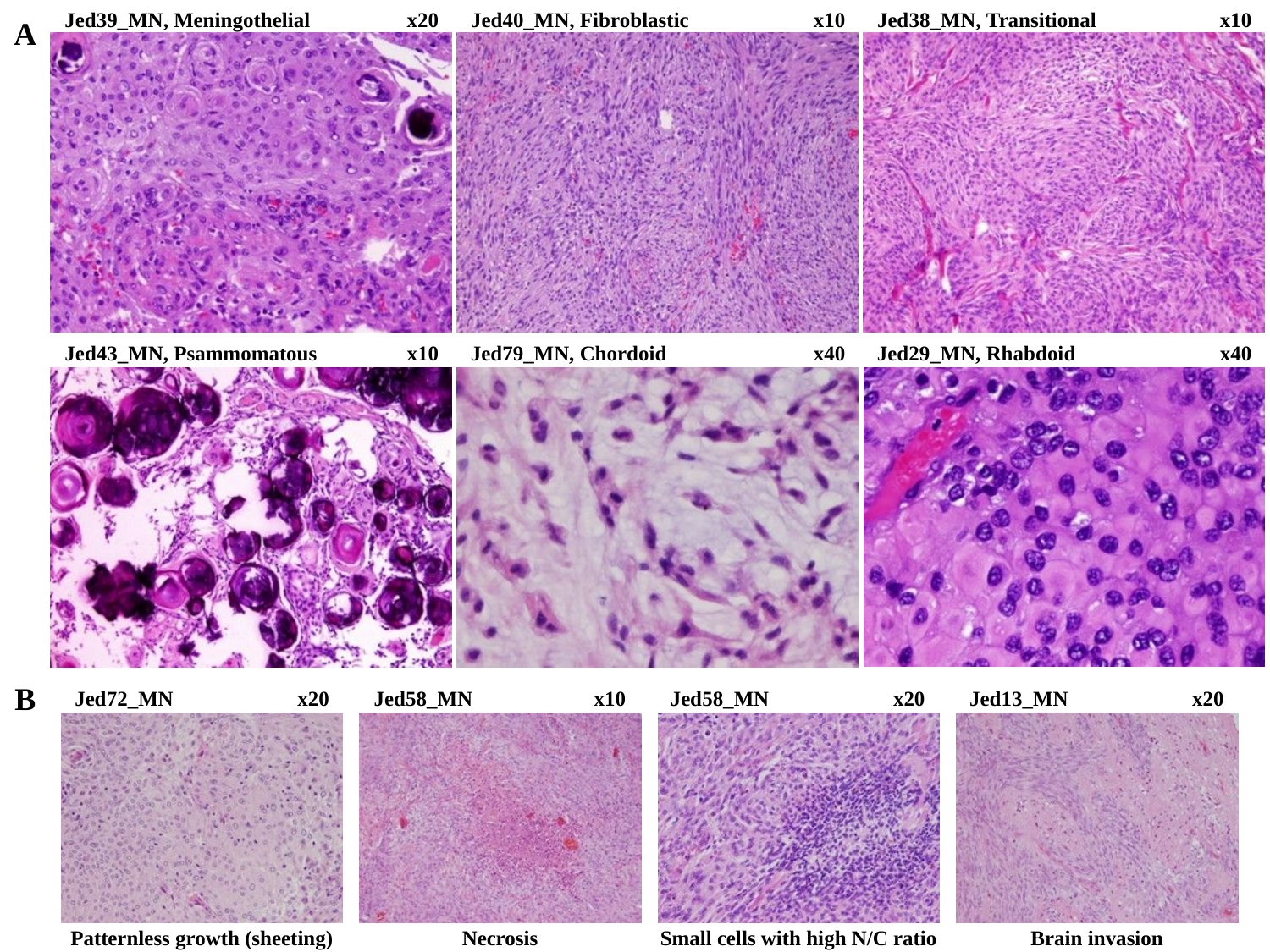

Jed39_MN, Meningothelial
x20
Jed40_MN, Fibroblastic
x10
Jed38_MN, Transitional
x10
A
Jed43_MN, Psammomatous
x10
Jed79_MN, Chordoid
x40
Jed29_MN, Rhabdoid
x40
B
Jed72_MN
x20
Jed58_MN
x10
Jed58_MN
x20
Jed13_MN
x20
Patternless growth (sheeting)
Necrosis
Small cells with high N/C ratio
Brain invasion
