# Supplementary material for: In situ characterization of stem cells-like biomarkers in meningiomas
Source: Cancer Cell Int. 2018 May 25;18:77. doi: 10.1186/s12935-018-0571-6 (PMC5970464; doi:10.1186/s12935-018-0571-6)
Supplement: Supplementary file 3 — Additional file 3: Table S2. Differentially expressed cancer driver genes [66] in individual tumors compared with three normal brain sample data sets, referenced in Gene Expression Omnibus (GEO) submission GSE77259. Values were generated from previously published data sets [64, 65] using Transcriptome Analysis Console v. 4.0. [file 12935_2018_571_MOESM3_ESM.docx]

**Additional Table 2. Differentially expressed cancer driver genes (66) in individual tumors compared with three normal brain sample data sets, referenced in Gene Expression Omnibus (GEO) submission GSE77259. Values were generated from previously published data sets (64, 65) using Transcriptome Analysis Console v. 4.0.**

| Gene list | Gene Mechanism | Fold Change (FC) | | | | | | | | | |
| --- | --- | --- | --- | --- | --- | --- | --- | --- | --- | --- | --- |
|  |  | Jed13_MN | Jed36_MN | Jed38_MN | Jed40_MN | Jed43_MN | Jed49_MN | Jed61_MN | Jed64_MN | Jed70_MN | Jed79_MN |
| PTPRD | TSG | -19.81 | -38.99 | -12.49 | -27.77 |  | -19.55 | -53.47 | -36.58 | -49.41 | -9.54 |
| ERBB4 | ONC | -10.12 | -25.56 | -26.83 | -20.81 | -9.46 | -29.99 | -28.5 | -16.52 | -4.67 | -23.43 |
| KIF1A | TSG | -8.52 | -4.89 | -21.07 | -9.94 | -12.52 | -6.66 | -43.04 | -37.5 | -11.58 | -31.31 |
| ZCCHC12 | ONC | -10.9 | -11.5 | -8.78 | -14.37 | -7.56 | -11.82 | -12.59 | -10.39 | -7.81 | -8.79 |
| ERBB3 | ONC | -10.16 | -8.75 | -7.09 | -7.9 | -9.68 | -9.53 | -10.13 | -6.84 | -9.14 | -8.56 |
| FBXW7 | TSG | -9.03 | -5.79 | -12.15 | -7.02 | -8.09 | -7.69 | -6.92 | -11.1 | -7.25 | -4.98 |
| CACNA1A | ◇ONC | -4.88 | -4.68 | -5.65 | -7.01 | -6.6 | -7.08 | -8.09 | -7.39 | -6.77 | -6.46 |
| KRT222 | ◇TSG |  | -12.53 | -9.09 | -6.9 |  | -3.56 | -13.17 | -9.7 | -9.42 |  |
| FGFR2 | ONC | -3.61 |  | -4.83 | -7.23 | -4.47 |  | -6.47 | -3.8 | -4.18 | -16.86 |
| MAP2K4 | TSG | -3.8 | -3.44 | -2.87 | -3.44 | -3.84 | -3.05 | -3.06 | -2.45 | -3.99 | -2.95 |
| MAP2K1 | ONC | -2.39 | -3.33 | -4.7 |  | -3.25 | -3.76 | -3.1 | -4.23 | -3.75 | -2.99 |
| DACH1 | ONC | -3.42 | -2.91 | -2.87 | -3.98 | -3.17 | -2.82 | -3.33 | -2.83 | -2.55 | -2.83 |
| IDH2 | ONC |  | -3.6 | -4.54 | -3.16 | -2.68 | -2.46 | -3.21 | -3.98 |  | -3.77 |
| NF2 | TSG | -2.11 | -2.28 |  | -2.76 |  | -3.02 | -3.3 |  | -2.28 |  |
| APC | TSG |  |  | -5.1 |  |  | -4.24 |  |  | -5.33 |  |
| MSH2 | TSG | -2.46 |  | -3.28 |  |  | -2.24 |  | -3.98 | -2.43 |  |
| ACVR2A | ◇TSG | -2.82 |  | -3.38 |  |  | -2.84 |  |  |  | -2.7 |
| PDGFRA | ◇ONC |  |  | -4.19 |  |  |  | -5.41 | -5.22 |  | 3.66 |
| PIK3CB | ◇ONC |  |  | -2.64 |  |  |  |  |  | -3.37 | -2.65 |
| SPTAN1 | TSG |  |  |  |  |  | -2.98 |  |  | -2.62 | -2.84 |
| ATXN3 | ◇TSG | -2.13 | -3.77 |  |  |  |  |  |  | -2.07 |  |
| MAPK1 | ONC |  | -2.58 | -2.91 |  | -2.39 |  |  |  |  |  |
| RET | ONC |  |  |  | -2.36 |  | -2.49 | -2.57 |  |  |  |
| EGR3 | ◇TSG |  |  |  |  | -3.52 |  | -3.74 |  |  |  |
| BRAF | ONC |  |  |  |  | -2.38 |  |  |  | -2.34 | -2.23 |
| NFE2L2 | ONC |  |  | -2.31 |  |  |  |  |  |  | -3.09 |
| PSIP1 | TSG |  |  |  |  |  | -3.1 |  |  |  | -2.28 |
| KIT | ONC |  |  | -4.94 | -3.94 |  | 3.51 |  |  |  |  |
| PMS2 | ◇TSG |  |  |  |  |  |  |  | -2.58 | -2.63 |  |
| RHOB | ONC |  | -2.52 |  |  | -2.22 |  |  |  |  |  |
| POLRMT | ONC |  |  |  |  | -2.37 |  | -2.22 |  |  |  |
| GNAQ | ONC |  |  | -2.22 |  |  |  |  |  |  | -2.17 |
| EIF1AX | ◇ONC |  |  | -3.57 |  |  |  |  |  |  |  |
| DMD | ONC |  | 2.37 | -2.33 |  |  |  | 2.1 |  |  | -5.14 |
| ARID5B | ◇TSG |  | -2.97 |  |  |  |  |  |  |  |  |
| AMER1 | ◇TSG |  |  |  | -2.84 |  |  |  |  |  |  |
| FLT3 | ONC |  |  |  |  |  |  |  |  |  | -2.75 |
| MACF1 | ◇ONC |  |  |  |  |  | -2.51 |  |  |  |  |
| RAD21 | ◇ONC | -2.51 |  |  |  |  |  |  |  |  |  |
| MYCN | ONC |  |  |  | -2.2 |  |  |  |  |  |  |
| GNA13 | ONC |  |  | -2.17 |  |  |  |  |  |  |  |
| EPHA3 | ONC |  | 3.12 | -2.55 | -2.57 |  |  |  |  |  |  |
| PIK3R1 | ◇TSG |  |  |  | 2.45 |  | -2.14 |  |  |  |  |
| KMT2C | TSG |  |  |  |  |  |  | 2.08 |  |  |  |
| SMC1A | ONC |  |  |  |  |  |  |  | 2.11 |  |  |
| RHOA | ONC |  |  |  |  |  |  | 2.13 |  |  |  |
| RFC1 | ◇TSG |  |  |  | 2.18 |  |  |  |  |  |  |
| ASXL1 | TSG |  |  |  |  |  |  |  |  | 2.4 |  |
| ASXL2 | ◇TSG |  | 2.41 |  |  |  |  |  |  |  |  |
| RAF1 | ONC |  |  |  | 2.52 |  |  |  |  |  |  |
| PLCB4 | ONC |  |  | 3.23 |  | 2.95 |  | -3.39 |  |  |  |
| PTCH1 | TSG |  |  | 2.79 |  |  |  |  |  |  |  |
| SMC3 | ◇ONC |  |  |  |  |  |  |  |  |  | 2.83 |
| PPM1D | ONC |  |  |  |  |  | 2.88 |  |  |  |  |
| JAK1 | TSG |  |  |  |  |  |  |  |  |  | 2.92 |
| MECOM | ◇ONC |  | 3.02 |  |  |  |  |  |  |  |  |
| GATA3 | ◇TSG |  | 3.04 |  |  |  |  |  |  |  |  |
| COL5A1 | ONC |  |  |  |  |  |  | 3.6 |  |  |  |
| CHD3 | ◇TSG |  |  |  |  |  |  |  | 3.64 |  |  |
| SMAD4 | ◇TSG |  |  |  |  |  |  | 4.35 |  |  |  |
| BCOR | TSG | 2.29 |  |  |  | 2.24 |  |  |  |  |  |
| H3F3C | Unknown |  |  | 2.65 |  |  |  | 2.36 |  |  |  |
| SMARCB1 | ◇ONC |  |  |  |  |  | 2.12 | 2.99 |  |  |  |
| DIAPH2 | ◇ONC |  |  | 2.74 |  |  |  | 2.42 |  |  |  |
| PLCG1 | ONC |  |  | 2.32 |  |  |  |  |  | 3 |  |
| MYH9 | ONC |  |  | 5.33 |  |  |  |  |  |  |  |
| MAP3K1 | TSG |  |  |  |  |  |  | 5.81 |  |  |  |
| FAT1 | TSG |  |  |  |  |  |  |  |  |  | 6.25 |
| TCF7L2 | TSG |  |  |  |  |  |  | 2.93 |  |  | 3.54 |
| BCL2L11 | UCS |  |  |  |  |  |  | 2.24 |  | 2.02 | 2.38 |
| SMAD2 | ◇TSG |  |  |  |  |  |  | 3.99 | 2.82 |  |  |
| ERCC2 | ONC | 3.5 |  |  |  |  | 3.44 |  |  |  |  |
| SOS1 | ONC |  | 2.3 |  |  |  |  | 2.63 |  |  | 2.24 |
| CDKN1B | TSG |  |  |  | 4.31 | 3.65 |  |  |  |  |  |
| RARA | ONC | 2.35 |  |  | 2.22 |  |  | 3.49 |  |  |  |
| ATF7IP | TSG |  | 2.96 |  |  |  |  | 2.76 | 2.48 |  |  |
| TGFBR2 | ◇TSG |  |  |  |  |  |  |  |  |  | 8.31 |
| HIST1H1C | Unknown |  |  |  |  |  | 5.71 |  | 3.08 |  |  |
| ATM | TSG |  | 2.15 |  | 2.07 | 2.42 |  | 2.32 |  |  |  |
| MLH1 | TSG | 2.26 | 2.21 |  |  | 2.27 | 2.75 |  |  |  |  |
| BRCA2 | ◇TSG |  |  |  |  |  | 2.22 | 3.58 |  | 2.51 | 2.22 |
| FGFR1 | ONC | 2.95 |  |  |  |  |  | 3.86 |  |  | 3.85 |
| STAG2 | TSG |  |  |  | 2.83 | 2.61 |  | 2.44 | 3.04 |  |  |
| TLR4 | ◇ONC | |  |  |  |  |  |  | 12.06 |  |  |
| RQCD1 | ◇ONC |  | 2.39 |  | 2.16 | 2.36 |  | 2.99 |  | 2.25 |  |
| ZFP36L1 | ◇TSG |  |  |  |  |  |  | 4.23 | 2.56 | 2.79 | 2.59 |
| MYC | ONC |  |  |  |  |  |  |  | 13.35 |  |  |
| KDM6A | TSG |  |  |  | 3.11 | 2.84 |  | 2.92 | 2.6 | 2.15 |  |
| ESR1 | ◇ONC | 2.26 | 2.57 |  |  | 2.56 | 2.56 | 2.11 |  | 2.38 |  |
| CDKN1A | TSG |  |  |  |  |  | 3.86 | 5.38 |  | 5.95 |  |
| RB1 | TSG |  | 2.92 |  | 2.88 | 3.28 | 3.05 | 3.49 |  |  |  |
| CARD11 | ◇ONC | 2.75 | 2.96 |  |  | 2.55 | 2.31 | 2.65 |  | 2.67 |  |
| IL7R | ONC |  |  |  | 2.68 | 3.64 | 3.54 | 3.4 |  | 2.92 |  |
| ABL1 | TSG | 2.39 |  | 2.41 | 3 | 2.2 | 2.14 | 2.39 | 2.57 |  |  |
| NOTCH2 | ONC | 3.41 |  |  |  | 3.59 |  | 3.21 | 3.02 |  | 4.39 |
| CDKN2A | ◇TSG |  |  |  |  |  | 5.13 |  |  | 6.35 | 6.91 |
| FGFR3 | ONC | 3.11 | 2.39 | 2.24 |  |  | 3.29 |  | 4.32 |  | 3.99 |
| GNA11 | ONC | 3.05 | 2.5 | 2.72 | 2.23 |  | 2.21 |  | 2.69 | 2.06 | 3.04 |
| JAK2 | TSG | 3.9 |  | 9.77 | 6.91 |  |  |  |  |  |  |
| EEF2 | TSG | 2.28 | 2.54 | 2.04 | 2.52 | 2.29 | 2.52 |  | 2.44 | 2.13 | 2.15 |
| RXRA | ONC | 3.35 | 2.96 | 3.37 |  |  | 3.04 | 2.98 |  | 2.95 | 3.01 |
| SETBP1 | ◇ONC | 2.65 |  | 3.57 | 2.89 | 2.36 |  | 3.87 | 3.16 |  | 4.32 |
| MED12 | ONC | 2.75 | 2.67 | 2.33 | 2.75 | 2.38 | 2.26 | 2.52 | 2.88 | 2.8 | 2.37 |
| AR | ONC | 6.5 |  | 2.83 | 9.74 | 6.86 |  |  |  |  |  |
| IDH1 | ONC | 3.02 | 2.75 |  | 2.75 | 4.63 | 2.7 | 3.32 | 2.63 | 3.03 | 2.46 |
| ACVR1 | ONC | 2.82 | 2.66 |  | 2.47 | 2.68 | 2.87 | 3.58 | 3.76 | 2.66 | 4.4 |
| ARID2 | ◇TSG | 3.01 | 3.08 | 3.31 | 3.58 | 4.02 |  | 3.44 | 3.89 |  | 3.88 |
| AKT1 | ONC | 3.55 | 2.11 | 2.23 | 3.82 | 3.07 | 2.87 | 2.29 | 2.36 | 3.04 | 3.68 |
| APOB | ◇TSG | 4.77 | 3.24 |  | 3.42 | 7.27 |  |  | 4.72 |  | 7.07 |
| ZBTB20 | ◇TSG | 2.8 | 4.35 | 2.78 | 3.01 | 4.13 |  | 3.37 | 3.54 | 3.6 | 2.91 |
| ARAF | unknown | 3.31 | 3.56 |  | 2.94 | 2.46 | 4.7 | 3.4 | 4 | 4.1 | 3.04 |
| TCF12 | ◇TSG | 3.21 | 3.51 | 2.34 | 3.49 | 2.95 | 3.16 | 4.34 | 2.8 | 3.33 | 2.52 |
| HIST1H1E | dual | 3.59 |  |  | 3.8 | 3.5 | 3.64 | 5.7 | 3.81 | 4.96 | 3.41 |
| CDK12 | TSG | 2.85 | 3.49 | 3.38 | 2.34 | 2.86 | 3.85 | 4.89 | 3.06 | 2.87 | 2.85 |
| TET2 | Unknown | 2.72 | 3.55 | 3.74 | 3.52 | 3.65 | 3.07 | 4.77 | 2.52 | 3.07 | 2.58 |
| PTPRC | ◇ONC | 6.67 |  |  | 6.01 | 6.8 | 4.54 | 5.16 |  | 6.14 |  |
| INPPL1 | ◇TSG | 3.88 | 3.6 | 5.09 | 3.52 | 3.85 | 3.12 | 3.16 | 3 | 3.62 | 3.12 |
| PIK3CG | ◇ONC | 6.92 | 2.65 |  | 5.38 | 6 | 3.98 | 5.01 | 2.84 | 4.42 |  |
| FLNA | TSG | 5.29 |  | 5.13 | 4.7 | 4.79 |  | 4.38 | 4.9 |  | 8.29 |
| CDK4 | ONC | 4 | 3.46 | 3.61 | 3.46 | 3.19 | 4.5 | 4.11 | 3.46 | 4.95 | 4.15 |
| LATS2 | TSG | 3.82 | 4.29 | 3.45 | 3.77 | 3.77 | 5.13 | 4.64 | 3.81 | 4.2 | 4.76 |
| PLXNB2 | ◇ONC | 5.09 | 4.33 | 3.94 | 3.42 | 4.17 | 3 | 4.44 | 3.66 | 4.59 | 5.97 |
| ZFHX3 | ◇TSG | 5.42 | 6.78 | 5.71 | 7.42 | 5.4 | 2.82 | 6.42 | 5.07 | 4.23 | 5.28 |
| AJUBA | TSG | 9.56 | 4.28 | 3.88 | 4.95 | 5.41 | 6.69 | 7.5 | 8.38 | 3.84 | 2.68 |
| CASP8 | ◇TSG | 4.77 | 7.29 | 3.8 | 5.02 | 6.06 | 6.97 | 7.99 | 5.02 | 5.02 | 5.73 |
| ERBB2 | ONC | 7.59 | 5.63 | 6.03 | 5.19 | 6.83 | 5.43 | 6.86 | 6.2 | 5.63 | 6.34 |
| TP53 | TSG | 7.36 | 9.85 | 3.63 | 5.82 | 5.39 | 8.16 | 7.51 | 8.72 | 5.48 | 7.45 |
| MET | dual |  | 13.24 | 3.71 | 3.6 | 6.6 | 6.32 | 10.11 | 6.08 | 7.59 | 13.81 |
| EGFR | ONC | 6.82 | 12.01 | 7.09 | 5.52 | 7.07 | 6.48 | 8.55 | 5.39 | 6.63 | 6.66 |
| PGR | ◇TSG | 3.6 |  | 18.35 | 5.12 | 8.12 | 8.84 |  | 30.57 |  | 9.57 |
| TNFAIP3 | TSG | 8.9 | 6.15 | 3.47 | 8.89 | 7.68 | 8.45 | 7.37 | 7.35 | 11.4 | 22.95 |
| ZFP36L2 | ◇TSG | 10.18 | 10.49 | 10.39 | 9.42 | 6.62 | 9.14 | 11.97 | 8.56 | 12.71 | 5.81 |
| BTG2 | ◇TSG | 7.96 | 9.16 | 5.79 | 14.65 | 4.73 | 12.6 | 5.42 | 14.62 | 16.06 | 9.19 |
| MYD88 | ONC | 13.94 | 11.15 | 4.92 | 13.48 | 14.55 | 9.33 | 9.15 | 8.39 | 9.46 | 9.46 |
| CCND1 | ◇ONC | 13.33 | 22.32 | 12.66 | 11.87 | 6.64 | 18.46 | 13.53 | 11.97 | 6.33 | 15.89 |
| KLF5 | ONC | 13.29 | 24.69 | 20.21 | 13.01 | 18.25 | 15.34 | 9.95 | 27.34 | 16.93 | 16.53 |
| CDH1 | TSG | 26.01 | 49.73 | 27.38 | 18.55 | 18.42 | 36.09 | 35.16 | 24.25 | 25.38 | 13.6 |

Gene mechanism prediction was based on previous publication (66). ◇: Possible; ONC: Oncogene; TSG: Tumor Suppressor Gene.
