# Supplementary material for: In situ characterization of stem cells-like biomarkers in meningiomas
Source: Cancer Cell Int. 2018 May 25;18:77. doi: 10.1186/s12935-018-0571-6 (PMC5970464; doi:10.1186/s12935-018-0571-6)
Supplement: Supplementary file 4 — Additional file 4: Figure S2. A bar graph showing averages of counts for Ki67 stained sections collected using manual counting or automated counting in Image J software. [file 12935_2018_571_MOESM4_ESM.pptx]

## Slide 1
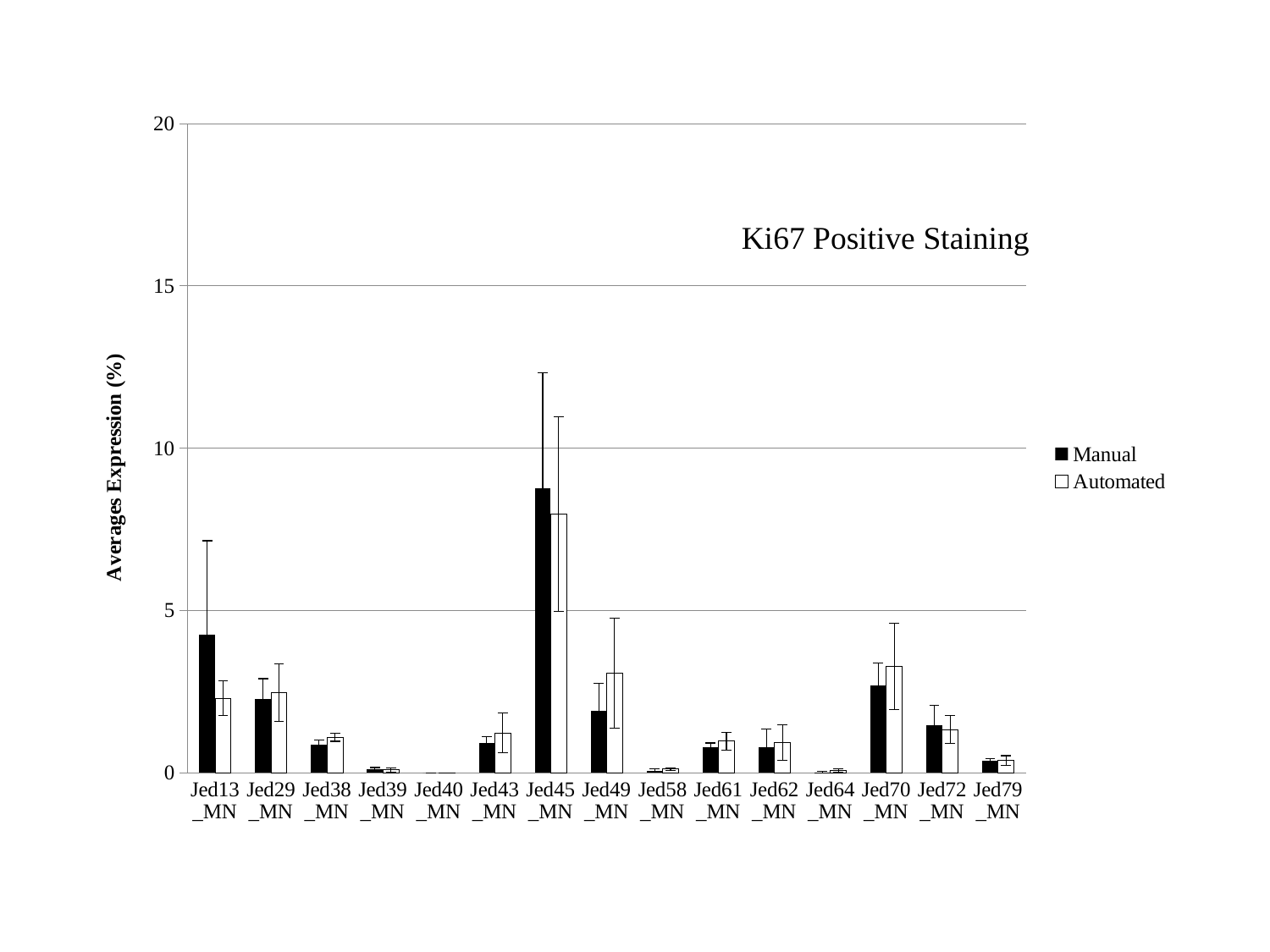

### Chart
| Category | Manual | Automated |
|---|---|---|
| Jed13_MN | 4.254166767337477 | 2.299490020473706 |
| Jed29_MN | 2.2671604111486454 | 2.469406864899797 |
| Jed38_MN | 0.8805537010546824 | 1.0908139521745057 |
| Jed39_MN | 0.10207271404967114 | 0.08733624454148471 |
| Jed40_MN | 0.0 | 0.0 |
| Jed43_MN | 0.9237044467439086 | 1.2229630930917694 |
| Jed45_MN | 8.754770572436332 | 7.972657023535893 |
| Jed49_MN | 1.8995896589173902 | 3.0692873488359163 |
| Jed58_MN | 0.07019309602183037 | 0.11489533740134368 |
| Jed61_MN | 0.7796094846739607 | 0.972913143750359 |
| Jed62_MN | 0.7824644099227079 | 0.9407686167497694 |
| Jed64_MN | 0.0213903743315508 | 0.070914667354602 |
| Jed70_MN | 2.703242330953924 | 3.2846693331621033 |
| Jed72_MN | 1.4660027858205882 | 1.3340200064726824 |
| Jed79_MN | 0.36113049580871603 | 0.37504256950381565 |Ki67 Positive Staining
