# Supplementary material for: In situ characterization of stem cells-like biomarkers in meningiomas
Source: Cancer Cell Int. 2018 May 25;18:77. doi: 10.1186/s12935-018-0571-6 (PMC5970464; doi:10.1186/s12935-018-0571-6)
Supplement: Supplementary file 6 — Additional file 6: Figure S4. A. Representative immunofluorescence images for consecutive sections for the grade III Jed29_MN meningioma. Sections were double stained for Ki67 (red) with Nestin (green), SOX2 (red) with CD133 (green), Vimentin (green) with FZD9 (red), SSEA4 (green) with SOX2 (red), and SSEA4 (green) with Olig2 (red), and each section was stained with DAPI (blue). Single staining of GFAP (red) or BIIITubulin (red) is also shown. All images were taken at 20x. B. A grid used as a repository of information for categorical staining is shown with a color-coded legend and size dimensions for sub-areas. [file 12935_2018_571_MOESM6_ESM.pptx]

## Slide 1
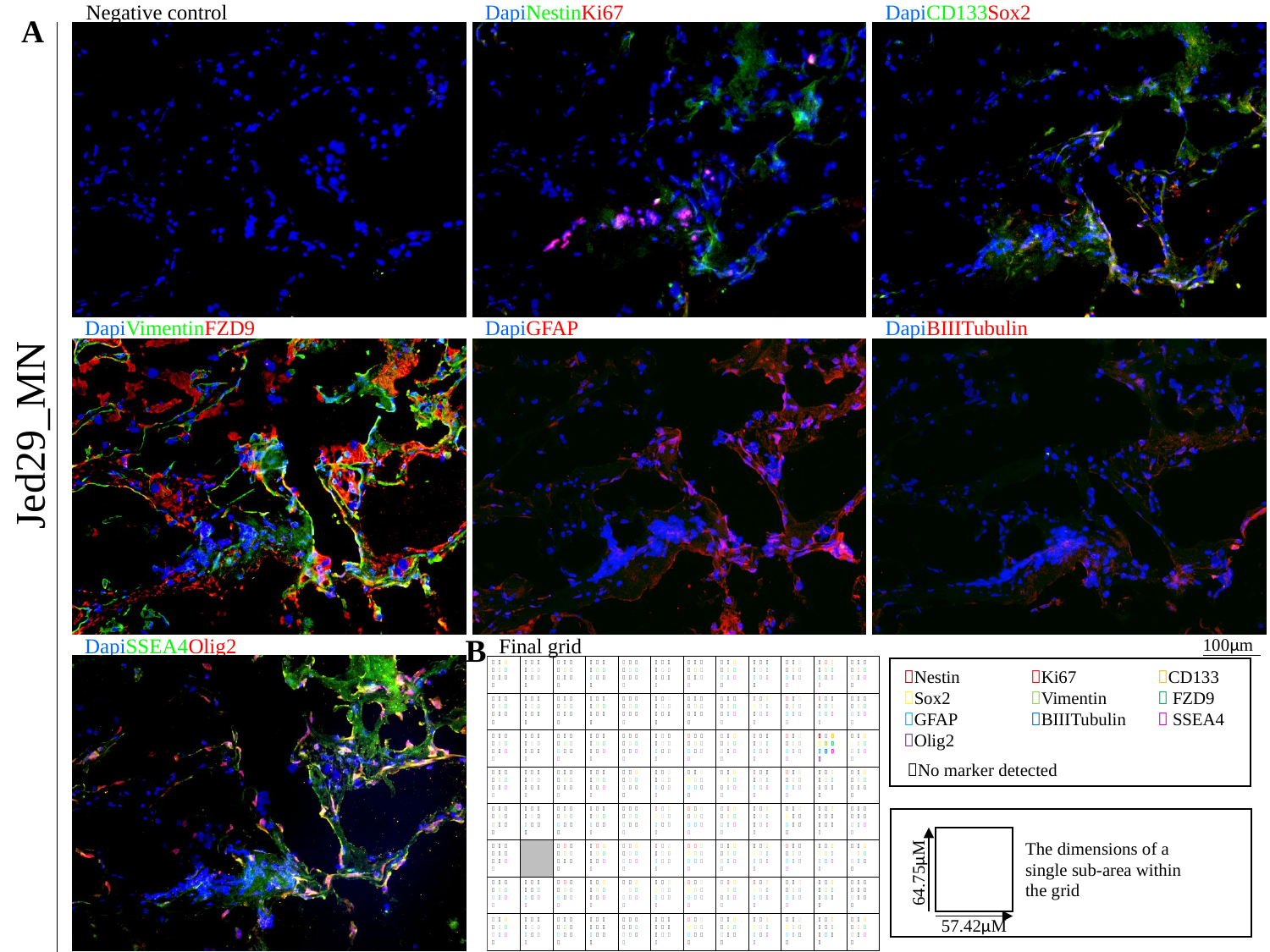

Negative control
DapiNestinKi67
DapiCD133Sox2
A
DapiVimentinFZD9
DapiGFAP
DapiBIIITubulin
Jed29_MN
B
DapiSSEA4Olig2
Final grid
100µm
Nestin 	Ki67	CD133
Sox2	Vimentin	 FZD9
GFAP	BIIITubulin	 SSEA4
Olig2
No marker detected
64.75µM
57.42µM
The dimensions of a single sub-area within the grid
