# Supplementary material for: In situ characterization of stem cells-like biomarkers in meningiomas
Source: Cancer Cell Int. 2018 May 25;18:77. doi: 10.1186/s12935-018-0571-6 (PMC5970464; doi:10.1186/s12935-018-0571-6)
Supplement: Supplementary file 7 — Additional file 7: Table S3. All combinations of markers observed in consecutive sections and their frequencies in all 15 meningioma samples. [file 12935_2018_571_MOESM7_ESM.docx]

**Additional Table 3.** **All combinations of markers observed in consecutive sections and their frequencies in all 15 meningioma samples.**

| **Markers in each Sub region** | **Qualitative Occurrence** | | | | | | | | | | | | | | | | | |
| --- | --- | --- | --- | --- | --- | --- | --- | --- | --- | --- | --- | --- | --- | --- | --- | --- | --- | --- |
|  | **Grade I** | | | | | | | | | **Grade II/III** | | | | | | | | **Total** |
|  | **Jed38_MN** | **Jed39_MN** | **Jed40_MN** | **Jed43_MN** | **Jed61_MN** | **Jed62_MN** | **Jed64_MN** | **Jed70_MN** | **Total** | **Jed13_MN** | **Jed29 _MN** | **Jed45_MN** | **Jed49_MN** | **Jed58_MN** | **Jed72_MN** | **Jed79_MN** | **Total** |  |
| VGEO |  |  |  | 6 | 27 | 2 | 30 |  | 65 | 22 |  | 1 | 2 |  | 2 | 3 | 30 | 95 |
| VEO |  | 2 |  | 8 | 4 | 9 | 30 |  | 53 | 7 |  | 2 |  |  | 2 |  | 11 | 64 |
| VGBEO |  |  | 1 |  | 43 | 5 |  |  | 49 |  |  | 3 |  | 1 |  | 4 | 8 | 57 |
| NCVGEO | 2 | 3 |  |  |  |  |  | 20 | 25 |  |  | 3 |  | 11 | 17 |  | 31 | 56 |
| NCSVFGEO | 24 | 2 |  |  |  |  |  | 6 | 32 | 2 |  | 2 | 2 |  |  |  | 6 | 38 |
| EV | 1 | 2 |  | 22 |  | 1 | 1 |  | 27 |  |  | 5 |  |  |  | 1 | 6 | 33 |
| NVGEO | 8 |  |  | 3 |  | 2 |  |  | 13 | 7 |  | 1 | 1 | 6 | 5 |  | 20 | 33 |
| CSVGBEO |  |  | 29 |  |  |  |  |  | 29 |  |  |  |  |  |  | 4 | 4 | 33 |
| NCVFGEO | 1 | 3 |  |  |  |  |  | 14 | 18 | 2 | 1 | 1 | 1 | 5 | 2 |  | 12 | 30 |
| VGE |  |  | 4 | 10 |  | 6 | 1 |  | 21 | 1 |  | 2 |  | 1 |  | 1 | 5 | 26 |
| NCSVGEO | 10 | 2 | 1 |  |  |  |  | 4 | 17 | 5 |  |  |  |  | 4 |  | 9 | 26 |
| NCVGBEO |  | 5 |  |  |  |  |  | 6 | 11 |  |  | 11 |  | 2 | 1 |  | 14 | 25 |
| NCVFGBEO |  | 5 |  |  |  |  |  | 6 | 11 |  | 5 | 4 |  | 5 |  |  | 14 | 25 |
| E | 1 |  | 1 | 2 | 1 | 2 |  |  | 7 |  |  | 7 | 8 | 1 | 1 |  | 17 | 24 |
| NVFGEO | 8 | 2 |  | 2 |  |  |  | 2 | 14 | 7 | 1 |  | 1 |  | 1 |  | 10 | 24 |
| V |  |  | 1 | 9 |  | 8 | 3 |  | 21 |  |  | 1 |  |  |  | 1 | 2 | 23 |
| VFE | 2 |  |  | 10 |  |  |  |  | 12 |  | 1 | 2 | 6 |  |  |  | 9 | 21 |
| NVE | 1 |  |  | 1 |  |  |  |  | 2 |  |  |  |  |  | 18 |  | 18 | 20 |
| CVEO |  | 10 |  |  |  |  | 5 |  | 15 | 1 |  | 1 | 1 |  |  | 2 | 5 | 20 |
| NVEO | 1 | 1 |  |  |  | 2 |  |  | 4 |  |  | 1 |  |  | 14 |  | 15 | 19 |
| CVGBEO |  |  | 1 |  | 1 |  |  |  | 2 |  |  | 1 |  |  |  | 16 | 17 | 19 |
| VFEO |  |  |  | 1 |  | 1 |  |  | 2 | 1 | 6 |  | 9 |  |  |  | 16 | 18 |
| CVGEO |  |  | 1 |  |  |  | 10 |  | 11 | 1 |  | 1 |  | 1 |  | 3 | 6 | 17 |
| NCSVGBEO |  | 1 | 2 |  |  |  |  | 8 | 11 | 1 |  | 4 |  |  | 1 |  | 6 | 17 |
| CSVFGBEO |  |  |  |  |  |  |  |  | 0 |  | 5 |  | 5 | 2 |  | 4 | 16 | 16 |
| NG |  |  |  |  |  | 2 |  |  | 2 |  |  |  |  | 11 |  |  | 11 | 13 |
| VG |  |  |  |  | 1 | 3 | 1 |  | 5 |  | 3 |  |  | 3 |  | 2 | 8 | 13 |
| NVGE | 3 |  |  | 1 |  |  |  |  | 4 | 1 |  |  |  | 5 | 3 |  | 9 | 13 |
| GE |  |  | 6 | 1 |  | 4 |  |  | 11 |  |  |  |  | 1 |  |  | 1 | 12 |
| VGBE |  |  |  |  | 3 | 6 |  |  | 9 |  |  | 2 |  |  |  | 1 | 3 | 12 |
| NCVGE | 3 | 1 |  |  |  |  |  | 4 | 8 |  |  |  |  | 3 |  |  | 3 | 11 |
| NVFGE | 9 |  |  |  |  |  |  |  | 9 |  |  |  | 1 |  | 1 |  | 2 | 11 |
| G |  |  |  |  |  | 8 |  |  | 8 |  | 2 |  |  |  |  |  | 2 | 10 |
| VF |  |  |  | 2 |  |  |  |  | 2 |  | 6 |  | 2 |  |  |  | 8 | 10 |
| VFGEO | 1 |  |  | 2 |  |  |  |  | 3 | 2 | 1 |  | 2 |  | 1 | 1 | 7 | 10 |
| CSVFGEO | 1 |  |  |  |  |  |  |  | 1 | 1 | 1 |  | 7 |  |  |  | 9 | 10 |
| NCSVFGBEO | 1 | 2 |  |  |  |  |  |  | 3 | 3 | 3 |  |  |  | 1 |  | 7 | 10 |
| CSVGEO |  |  | 7 |  |  |  | 1 |  | 8 | 1 |  |  |  |  |  |  | 1 | 9 |
| VFGBEO |  |  |  |  | 1 | 2 |  |  | 3 |  | 2 | 2 | 1 |  |  | 1 | 6 | 9 |
| CVFGBEO |  |  |  |  |  |  |  |  | 0 |  | 1 | 1 |  | 1 |  | 6 | 9 | 9 |
| Z |  |  |  |  |  | 8 |  |  | 8 |  |  |  |  |  |  |  | 0 | 8 |
| VBE |  | 1 |  |  |  | 2 |  |  | 3 |  |  | 3 |  |  |  | 2 | 5 | 8 |
| VFGE | 1 |  |  | 2 |  |  |  |  | 3 |  | 1 | 1 | 3 |  |  |  | 5 | 8 |
| NCVBE |  | 8 |  |  |  |  |  |  | 8 |  |  |  |  |  |  |  | 0 | 8 |
| SVGEO |  |  | 1 | 1 |  |  |  |  | 2 | 5 |  |  |  |  | 1 |  | 6 | 8 |
| NSVFGEO | 3 |  |  |  |  |  |  |  | 3 | 5 |  |  |  |  |  |  | 5 | 8 |
| EO |  | 2 |  |  |  | 2 |  |  | 4 |  | 1 |  | 2 |  |  |  | 3 | 7 |
| GEO |  | 1 |  | 1 |  |  | 3 |  | 5 | 1 |  |  | 1 |  |  |  | 2 | 7 |
| NCVEO |  | 5 |  |  |  |  |  |  | 5 | 1 |  |  |  |  | 1 |  | 2 | 7 |
| NVFGBEO |  |  |  |  |  | 1 |  |  | 1 | 1 | 4 | 1 |  |  |  |  | 6 | 7 |
| NKCVGBEO |  |  |  |  |  |  |  |  | 0 |  |  | 7 |  |  |  |  | 7 | 7 |
| NKCVFGBEO |  |  |  |  |  |  |  |  | 0 |  | 3 | 2 |  | 2 |  |  | 7 | 7 |
| NE |  | 2 |  |  |  |  |  |  | 2 |  |  |  |  |  | 4 |  | 4 | 6 |
| CVF |  |  |  |  |  |  |  |  | 0 |  | 4 |  | 2 |  |  |  | 6 | 6 |
| NVG |  |  |  |  |  | 2 |  |  | 2 |  |  |  |  | 4 |  |  | 4 | 6 |
| VBEO |  | 1 |  |  | 1 | 2 |  |  | 4 |  |  |  |  |  |  | 2 | 2 | 6 |
| CSVGE | 1 |  | 2 |  |  |  |  |  | 3 |  |  |  |  |  |  | 3 | 3 | 6 |
| CVFEO |  | 5 |  |  |  |  |  |  | 5 |  | 1 |  |  |  |  |  | 1 | 6 |
| CVGBE |  |  |  |  |  |  |  |  | 0 |  |  | 1 |  |  |  | 5 | 6 | 6 |
| NCSVGE |  |  |  |  |  |  |  | 6 | 6 |  |  |  |  |  |  |  | 0 | 6 |
| NCVFEO |  | 6 |  |  |  |  |  |  | 6 |  |  |  |  |  |  |  | 0 | 6 |
| NKCVGEO | 1 |  |  |  |  |  |  |  | 1 |  |  | 1 |  | 2 | 2 |  | 5 | 6 |
| BE |  |  |  |  |  | 1 |  |  | 1 |  |  | 2 | 1 |  |  | 1 | 4 | 5 |
| NGE |  |  |  |  |  |  |  |  | 0 |  |  |  |  | 4 | 1 |  | 5 | 5 |
| VFO |  |  |  |  |  |  |  |  | 0 |  | 5 |  |  |  |  |  | 5 | 5 |
| NCVE |  | 3 |  |  |  |  |  |  | 3 |  |  |  |  |  | 2 |  | 2 | 5 |
| SVFGEO | 1 |  |  |  |  |  |  |  | 1 | 4 |  |  |  |  |  |  | 4 | 5 |
| NSVGEO | 3 |  |  |  |  |  |  |  | 3 | 2 |  |  |  |  |  |  | 2 | 5 |
| CSVFEO |  | 2 |  |  |  |  |  |  | 2 |  |  |  | 3 |  |  |  | 3 | 5 |
| CVFBEO |  | 2 |  |  |  |  |  |  | 2 |  | 1 |  | 2 |  |  |  | 3 | 5 |
| B |  |  |  |  | 1 | 2 |  |  | 3 |  |  |  |  | 1 |  |  | 1 | 4 |
| NV |  | 1 |  | 1 |  |  |  | 2 | 4 |  |  |  |  |  |  |  | 0 | 4 |
| CSG |  |  | 2 |  |  |  |  |  | 2 |  | 1 |  |  | 1 |  |  | 2 | 4 |
| CVGO |  |  |  |  |  |  |  |  | 0 |  |  |  |  |  |  | 4 | 4 | 4 |
| GBEO |  |  |  |  |  |  |  |  | 0 |  | 1 |  |  |  |  | 3 | 4 | 4 |
| VFBEO |  |  |  |  |  |  |  |  | 0 |  |  | 1 | 2 |  |  | 1 | 4 | 4 |
| CVFGEO | 1 |  |  |  |  |  |  |  | 1 |  | 2 |  | 1 |  |  |  | 3 | 4 |
| KVGBEO |  |  |  |  | 4 |  |  |  | 4 |  |  |  |  |  |  |  | 0 | 4 |
| NVGBEO |  |  |  |  | 2 |  |  |  | 2 | 1 |  | 1 |  |  |  |  | 2 | 4 |
| NKCSVFGEO |  |  |  |  |  |  |  |  | 0 |  | 1 |  | 1 | 2 |  |  | 4 | 4 |
| N |  |  |  |  |  | 1 |  |  | 1 |  |  |  |  | 2 |  |  | 2 | 3 |
| CV |  |  | 1 |  |  |  | 2 |  | 3 |  |  |  |  |  |  |  | 0 | 3 |
| FE |  |  |  |  |  | 1 |  |  | 1 |  |  |  | 2 |  |  |  | 2 | 3 |
| BEO |  |  |  |  |  | 2 |  |  | 2 |  | 1 |  |  |  |  |  | 1 | 3 |
| GBE |  |  |  |  | 1 | 1 |  |  | 2 |  |  |  |  |  |  | 1 | 1 | 3 |
| NEO |  |  |  | 1 |  |  |  |  | 1 |  |  |  |  |  | 2 |  | 2 | 3 |
| VFG |  |  |  |  |  |  |  |  | 0 |  | 2 |  | 1 |  |  |  | 3 | 3 |
| CVFE |  | 1 |  |  |  |  |  |  | 1 | 1 |  | 1 |  |  |  |  | 2 | 3 |
| CVGE |  |  | 1 |  |  | 1 |  |  | 2 |  |  |  |  |  |  | 1 | 1 | 3 |
| VFGO |  |  |  |  |  |  |  |  | 0 |  | 3 |  |  |  |  |  | 3 | 3 |
| NSVGE | 2 |  |  |  |  |  |  |  | 2 | 1 |  |  |  |  |  |  | 1 | 3 |
| CSVEO |  |  | 2 |  |  |  | 1 |  | 3 |  |  |  |  |  |  |  | 0 | 3 |
| CVFGE |  |  |  |  |  |  |  |  | 0 |  | 1 |  | 1 |  |  | 1 | 3 | 3 |
| CSVBEO |  |  | 2 |  |  |  |  |  | 2 |  |  |  |  |  |  | 1 | 1 | 3 |
| CSVFGB |  |  |  |  |  |  |  |  | 0 |  | 3 |  |  |  |  |  | 3 | 3 |
| CVFGBE |  |  |  |  |  |  |  |  | 0 |  | 1 |  |  |  |  | 2 | 3 | 3 |
| NCVFGE |  |  |  |  |  |  |  | 1 | 1 |  |  |  |  | 2 |  |  | 2 | 3 |
| NCVGBE |  |  |  |  |  |  |  |  | 0 |  |  | 1 |  | 1 | 1 |  | 3 | 3 |
| NKVGEO |  |  |  |  |  |  |  |  | 0 | 1 |  |  |  |  | 2 |  | 3 | 3 |
| NVFBEO |  |  |  |  |  | 2 |  |  | 2 |  | 1 |  |  |  |  |  | 1 | 3 |
| CSVFBEO |  |  |  |  |  |  |  |  | 0 |  | 1 |  |  |  |  | 2 | 3 | 3 |
| NCVFBEO |  | 3 |  |  |  |  |  |  | 3 |  |  |  |  |  |  |  | 0 | 3 |
| NCVFGBE |  |  |  |  |  |  |  |  | 0 |  | 1 | 1 |  | 1 |  |  | 3 | 3 |
| NKCSVGEO | 1 |  |  |  |  |  |  |  | 1 | 1 |  |  |  |  | 1 |  | 2 | 3 |
| NSVFGBEO | 2 |  |  |  |  |  |  |  | 2 | 1 |  |  |  |  |  |  | 1 | 3 |
| NKCVFGEO |  |  |  |  |  |  |  | 1 | 1 | 1 |  |  |  | 1 |  |  | 2 | 3 |
| NKCSVGBEO |  |  |  |  |  |  |  | 1 | 1 |  |  | 2 |  |  |  |  | 2 | 3 |
| CE |  | 1 |  |  |  |  |  |  | 1 |  |  |  | 1 |  |  |  | 1 | 2 |
| GB |  |  |  |  |  |  |  |  | 0 |  |  |  |  | 1 |  | 1 | 2 | 2 |
| CEO |  | 2 |  |  |  |  |  |  | 2 |  |  |  |  |  |  |  | 0 | 2 |
| CVE |  | 1 |  |  |  |  |  |  | 1 |  |  | 1 |  |  |  |  | 1 | 2 |
| NVF |  |  |  |  |  | 1 |  | 1 | 2 |  |  |  |  |  |  |  | 0 | 2 |
| CGBE |  |  |  |  |  |  |  |  | 0 |  |  |  |  |  |  | 2 | 2 | 2 |
| CGEO |  |  |  |  |  |  | 1 |  | 1 |  |  |  |  |  | 1 |  | 1 | 2 |
| NVFE |  |  |  | 1 |  |  |  |  | 1 |  |  |  | 1 |  |  |  | 1 | 2 |
| NVFG |  |  |  |  |  |  |  | 1 | 1 |  |  |  | 1 |  |  |  | 1 | 2 |
| SVEO |  |  | 2 |  |  |  |  |  | 2 |  |  |  |  |  |  |  | 0 | 2 |
| CGBEO |  |  |  |  |  |  |  |  | 0 |  |  |  |  |  |  | 2 | 2 | 2 |
| CSGEO |  |  | 1 |  |  |  |  |  | 1 |  |  |  | 1 |  |  |  | 1 | 2 |
| CSVGB |  |  | 2 |  |  |  |  |  | 2 |  |  |  |  |  |  |  | 0 | 2 |
| KVGEO |  |  |  | 1 | 1 |  |  |  | 2 |  |  |  |  |  |  |  | 0 | 2 |
| NCVFG |  |  |  |  |  |  |  | 1 | 1 |  | 1 |  |  |  |  |  | 1 | 2 |
| NKVGE |  |  |  | 1 |  |  |  |  | 1 |  |  |  |  | 1 |  |  | 1 | 2 |
| NVFEO |  |  |  |  |  | 2 |  |  | 2 |  |  |  |  |  |  |  | 0 | 2 |
| VFGBE |  |  |  |  |  |  |  |  | 0 |  | 1 | 1 |  |  |  |  | 2 | 2 |
| CSGBEO |  |  | 2 |  |  |  |  |  | 2 |  |  |  |  |  |  |  | 0 | 2 |
| CSVGBE |  |  | 1 |  |  |  |  |  | 1 |  |  |  |  |  |  | 1 | 1 | 2 |
| KVFGEO |  |  |  |  |  |  |  |  | 0 |  | 2 |  |  |  |  |  | 2 | 2 |
| NCSVEO |  | 1 |  |  |  |  |  |  | 1 |  |  |  | 1 |  |  |  | 1 | 2 |
| NVFGBE |  |  |  |  |  |  |  |  | 0 |  |  | 1 | 1 |  |  |  | 2 | 2 |
| NKVFGEO | 1 |  |  |  |  |  |  |  | 1 | 1 |  |  |  |  |  |  | 1 | 2 |
| NCSVFEO |  | 1 |  |  |  |  |  |  | 1 |  |  |  | 1 |  |  |  | 1 | 2 |
| NKCVGBE |  |  |  |  |  |  |  |  | 0 |  |  | 2 |  |  |  |  | 2 | 2 |
| NCSFGBEO |  | 2 |  |  |  |  |  |  | 2 |  |  |  |  |  |  |  | 0 | 2 |
| NKCSVFGBEO | 1 |  |  |  |  |  |  |  | 1 |  | 1 |  |  |  |  |  | 1 | 2 |
| BO |  |  |  |  |  | 1 |  |  | 1 |  |  |  |  |  |  |  | 0 | 1 |
| CO |  |  |  |  |  |  |  |  | 0 |  | 1 |  |  |  |  |  | 1 | 1 |
| CS |  |  | 1 |  |  |  |  |  | 1 |  |  |  |  |  |  |  | 0 | 1 |
| SE |  |  | 1 |  |  |  |  |  | 1 |  |  |  |  |  |  |  | 0 | 1 |
| VO |  |  |  |  |  |  |  |  | 0 |  |  |  |  |  |  | 1 | 1 | 1 |
| CFB |  |  |  |  |  |  |  |  | 0 |  | 1 |  |  |  |  |  | 1 | 1 |
| CSE |  |  | 1 |  |  |  |  |  | 1 |  |  |  |  |  |  |  | 0 | 1 |
| CSV |  |  | 1 |  |  |  |  |  | 1 |  |  |  |  |  |  |  | 0 | 1 |
| CVB |  |  |  |  |  |  |  |  | 0 |  |  | 1 |  |  |  |  | 1 | 1 |
| CVO |  | 1 |  |  |  |  |  |  | 1 |  |  |  |  |  |  |  | 0 | 1 |
| KEO |  |  |  |  |  |  |  |  | 0 |  |  |  | 1 |  |  |  | 1 | 1 |
| KVF |  |  |  |  |  |  |  |  | 0 |  | 1 |  |  |  |  |  | 1 | 1 |
| NCG |  |  |  |  |  |  |  |  | 0 |  |  |  |  | 1 |  |  | 1 | 1 |
| NCV |  |  |  |  |  |  |  | 1 | 1 |  |  |  |  |  |  |  | 0 | 1 |
| SGE |  |  | 1 |  |  |  |  |  | 1 |  |  |  |  |  |  |  | 0 | 1 |
| SVG |  |  | 1 |  |  |  |  |  | 1 |  |  |  |  |  |  |  | 0 | 1 |
| VGB |  |  |  |  |  |  |  |  | 0 |  |  |  |  |  |  | 1 | 1 | 1 |
| CBEO |  |  |  |  |  |  |  |  | 0 |  |  |  |  |  |  | 1 | 1 | 1 |
| CGBO |  |  |  |  |  |  |  |  | 0 | 1 |  |  |  |  |  |  | 1 | 1 |
| CSEO |  |  | 1 |  |  |  |  |  | 1 |  |  |  |  |  |  |  | 0 | 1 |
| CSGB |  |  | 1 |  |  |  |  |  | 1 |  |  |  |  |  |  |  | 0 | 1 |
| CSGE |  |  | 1 |  |  |  |  |  | 1 |  |  |  |  |  |  |  | 0 | 1 |
| CSVG |  |  |  |  |  |  | 1 |  | 1 |  |  |  |  |  |  |  | 0 | 1 |
| CVBE |  |  |  |  |  |  |  |  | 0 |  |  |  |  |  |  | 1 | 1 | 1 |
| CVFB |  |  |  |  |  |  |  |  | 0 |  | 1 |  |  |  |  |  | 1 | 1 |
| CVFG |  |  |  |  |  |  |  |  | 0 |  | 1 |  |  |  |  |  | 1 | 1 |
| CVGB |  |  |  |  |  |  |  |  | 0 |  |  |  |  |  |  | 1 | 1 | 1 |
| FGBO |  |  |  |  |  |  |  |  | 0 |  | 1 |  |  |  |  |  | 1 | 1 |
| FGEO |  |  |  |  |  |  |  |  | 0 |  | 1 |  |  |  |  |  | 1 | 1 |
| KVFE |  |  |  |  |  |  |  |  | 0 |  |  |  | 1 |  |  |  | 1 | 1 |
| NCEO |  | 1 |  |  |  |  |  |  | 1 |  |  |  |  |  |  |  | 0 | 1 |
| NCVB |  |  |  |  |  |  |  |  | 0 |  |  | 1 |  |  |  |  | 1 | 1 |
| NKVE |  |  |  |  |  |  |  |  | 0 |  |  |  |  |  | 1 |  | 1 | 1 |
| NVFO |  |  |  |  |  |  |  |  | 0 |  | 1 |  |  |  |  |  | 1 | 1 |
| SVGB |  |  | 1 |  |  |  |  |  | 1 |  |  |  |  |  |  |  | 0 | 1 |
| SVGE |  |  | 1 |  |  |  |  |  | 1 |  |  |  |  |  |  |  | 0 | 1 |
| VGBO |  |  |  |  |  |  |  |  | 0 |  |  |  |  |  |  | 1 | 1 | 1 |
| CSGBE |  |  | 1 |  |  |  |  |  | 1 |  |  |  |  |  |  |  | 0 | 1 |
| CSVFE |  |  |  |  |  |  |  |  | 0 |  |  |  | 1 |  |  |  | 1 | 1 |
| CVBEO |  |  |  |  |  |  |  |  | 0 |  |  |  |  |  |  | 1 | 1 | 1 |
| CVGBO |  |  |  |  |  |  |  |  | 0 |  |  |  |  |  |  | 1 | 1 | 1 |
| KCVBE |  |  |  |  |  |  |  |  | 0 |  |  | 1 |  |  |  |  | 1 | 1 |
| KVFGE |  |  |  |  |  |  |  |  | 0 |  |  |  | 1 |  |  |  | 1 | 1 |
| NCFGE |  |  |  |  |  |  |  |  | 0 |  |  |  |  | 1 |  |  | 1 | 1 |
| NKCGE |  |  |  |  |  |  |  |  | 0 |  |  |  |  | 1 |  |  | 1 | 1 |
| NKCVE |  |  |  |  |  |  |  |  | 0 |  |  |  |  |  | 1 |  | 1 | 1 |
| NSVEO |  |  |  |  |  |  |  |  | 0 |  |  |  |  |  | 1 |  | 1 | 1 |
| NVBEO |  |  |  |  |  | 1 |  |  | 1 |  |  |  |  |  |  |  | 0 | 1 |
| NVGBE |  |  |  |  |  |  |  |  | 0 | 1 |  |  |  |  |  |  | 1 | 1 |
| SVBEO |  |  | 1 |  |  |  |  |  | 1 |  |  |  |  |  |  |  | 0 | 1 |
| SVFEO |  |  |  |  |  |  |  |  | 0 |  |  |  | 1 |  |  |  | 1 | 1 |
| CSVFGE |  |  |  |  |  |  |  |  | 0 |  |  |  | 1 |  |  |  | 1 | 1 |
| KCVFEO |  |  |  |  |  |  |  |  | 0 |  | 1 |  |  |  |  |  | 1 | 1 |
| KCVGBE |  |  |  |  |  |  |  |  | 0 |  |  | 1 |  |  |  |  | 1 | 1 |
| KCVGEO |  |  |  |  |  |  |  |  | 0 |  |  |  |  | 1 |  |  | 1 | 1 |
| NCGBEO |  |  |  |  |  |  |  |  | 0 |  |  |  |  |  | 1 |  | 1 | 1 |
| NCSBEO |  | 1 |  |  |  |  |  |  | 1 |  |  |  |  |  |  |  | 0 | 1 |
| NCSVFG |  |  |  |  |  |  |  |  | 0 |  | 1 |  |  |  |  |  | 1 | 1 |
| SVGBEO |  |  |  |  |  |  |  |  | 0 | 1 |  |  |  |  |  |  | 1 | 1 |
| CSVFGBE |  |  |  |  |  |  |  |  | 0 |  |  |  | 1 |  |  |  | 1 | 1 |
| KCVFGEO |  |  |  |  |  |  |  |  | 0 |  | 1 |  |  |  |  |  | 1 | 1 |
| KCVGBEO |  |  |  |  |  |  |  |  | 0 |  |  |  |  |  |  | 1 | 1 | 1 |
| NCSVBEO |  | 1 |  |  |  |  |  |  | 1 |  |  |  |  |  |  |  | 0 | 1 |
| NCSVGBE |  |  |  |  |  |  |  | 1 | 1 |  |  |  |  |  |  |  | 0 | 1 |
| NKCVFGE |  |  |  |  |  |  |  |  | 0 |  |  |  |  | 1 |  |  | 1 | 1 |
| NSVFBEO |  |  |  |  |  |  |  |  | 0 | 1 |  |  |  |  |  |  | 1 | 1 |
| SVFGBEO |  |  |  |  |  |  |  |  | 0 |  |  |  |  |  |  | 1 | 1 | 1 |
| KCSVFGBO |  |  |  |  |  |  |  |  | 0 |  |  |  |  |  |  | 1 | 1 | 1 |
| NCSVFBEO |  |  |  |  |  |  |  |  | 0 |  | 1 |  |  |  |  |  | 1 | 1 |
| NCSVFGBE |  |  |  |  |  |  |  |  | 0 |  |  | 1 |  |  |  |  | 1 | 1 |
| NKCVFGBE |  |  |  |  |  |  |  |  | 0 |  |  |  |  | 1 |  |  | 1 | 1 |
| NKSVGBEO |  |  |  |  |  |  |  |  | 0 |  |  | 1 |  |  |  |  | 1 | 1 |
| NKCSVFBEO |  |  |  |  |  |  |  |  | 0 |  | 1 |  |  |  |  |  | 1 | 1 |
| NKSVFGBEO |  |  |  |  |  |  |  |  | 0 |  |  | 1 |  |  |  |  | 1 | 1 |

Marker Symbols stand for, N: Nestin, K: Ki67, C: CD133, S: Sox2, V: Vimentin, F: FZD9, G: GFAP, B: BIII Tubulin, E: SSEA4, O: Olig2, Z: No Marker.
